# Supplementary material for: Construction of a new chromosome-scale, long-read reference genome assembly for the Syrian hamster, Mesocricetus auratus
Source: Gigascience. 2022 May 28;11:giac039. doi: 10.1093/gigascience/giac039 (PMC9155146; doi:10.1093/gigascience/giac039)
Supplement: giac039_GIGA-D-21-00197_Original_Submission [file giac039_giga-d-21-00197_original_submission.pdf]

GigaScience

Construction of a new chromosome-scale, long-read reference genome assembly of the Syrian hamster, Mesocricetus auratus

--Manuscript Draft--

**Manuscript Number:**	GIGA-D-21-00197
**Full Title:**	Construction of a new chromosome-scale, long-read reference genome assembly of the Syrian hamster, Mesocricetus auratus
**Article Type:**	Data Note
**Funding Information:**	division of intramural research, national institute of allergy and infectious diseases (HHSN272201600007C) — Dr David H. O'Connor
**Abstract:**	Background<br>The Syrian hamster (Mesocricetus auratus) has been suggested as a useful mammalian model for a variety of diseases and infections, including infection with respiratory viruses such as SARS-CoV-2. The MesAur1.0 genome assembly was published in 2013 using whole-genome shotgun sequencing with short-read sequence data. Current more advanced sequencing technologies and assembly methods now permit the generation of near-complete genome assemblies with higher quality and higher continuity.<br>Findings<br>Here, we report an improved assembly of the M. auratus genome (BCM_Maur_2.0) using Oxford Nanopore Technologies long-read sequencing to produce a chromosome-scale assembly. The total length of the new assembly is 2.46 Gbp, similar to the 2.50 Gbp length of a previous assembly of this genome, MesAur1.0. BCM_Maur_2.0 exhibits significantly improved continuity with a scaffold N50 that is 6.7 times greater than MesAur1.0. Furthermore, 21,616 protein coding genes and 10,459 noncoding genes were annotated in BCM_Maur_2.0 compared to 20,495 protein coding genes and 4,168 noncoding genes in MesAur1.0. This new assembly also improves the unresolved regions as measured by nucleotide ambiguities, where approximately 17.11% of bases in MesAur1.0 were unresolved compared to BCM_Maur_2.0 in which the number of unresolved bases is reduced to 3.00%.<br>Conclusions<br>Access to a more complete reference genome with improved accuracy and continuity will facilitate more detailed, comprehensive, and meaningful research results for a wide variety of future studies using Syrian hamsters as models.
**Corresponding Author:**	Jeffrey Rogers, Ph.D.<br>Baylor College of Medicine<br>Houston, Texas UNITED STATES
**Corresponding Author Secondary Information:**	
**Corresponding Author's Institution:**	Baylor College of Medicine
**Corresponding Author's Secondary Institution:**	
**First Author:**	R. Alan Harris
**First Author Secondary Information:**	
**Order of Authors:**	R. Alan Harris
	Muthuswamy Raveendran, Ph.D.,
	Dustin T Lyfoung
	Fritz J Sedlazeck
	Medhat Mahmoud
	Trent M Prall

Powered by Editorial Manager® and ProduXion Manager® from Aries Systems Corporation

	Julie A Karl
	Harshavardhan Doddapaneni
	Qingchang Meng
	Yi Han
	Donna Muzny
	Roger W Wiseman
	David H. O'Connor
	Jeffrey Rogers
**Order of Authors Secondary Information:**	
**Additional Information:**	
**Question**	**Response**
Are you submitting this manuscript to a special series or article collection?	No
**Experimental design and statistics**<br><br>Full details of the experimental design and statistical methods used should be given in the Methods section, as detailed in our Minimum Standards Reporting Checklist. Information essential to interpreting the data presented should be made available in the figure legends.<br><br>Have you included all the information requested in your manuscript?	Yes
**Resources**<br><br>A description of all resources used, including antibodies, cell lines, animals and software tools, with enough information to allow them to be uniquely identified, should be included in the Methods section. Authors are strongly encouraged to cite Research Resource Identifiers (RRIDs) for antibodies, model organisms and tools, where possible.<br><br>Have you included the information requested as detailed in our Minimum Standards Reporting Checklist?	Yes
**Availability of data and materials**	Yes

All datasets and code on which the conclusions of the paper rely must be either included in your submission or deposited in publicly available repositories (where available and ethically appropriate), referencing such data using a unique identifier in the references and in the "Availability of Data and Materials" section of your manuscript.<br><br>Have you have met the above requirement as detailed in our Minimum Standards Reporting Checklist?	

Powered by Editorial Manager® and ProduXion Manager® from Aries Systems Corporation

Main text Click here to access/download;Manuscript;Hamster_Genome_manuscript_To

Construction of a new chromosome-scale, long-read reference genome assembly of the Syrian hamster, *Mesocricetus auratus*

R. Alan Harris[1] rharris1@bcm.edu

Muthuswamy Raveendran[1] raveendr@bcm.edu

Dustin T. Lyfoung[2] lyfoung@wisc.edu

Fritz J Sedlazeck[1] fritz.sedlazeck@bcm.edu

Medhat Mahmoud[1] helmy.medhat@gmail.com

Trent M. Prall[3] prall@wisc.edu

Julie A. Karl[3] jakarl@wisc.edu

Harshavardhan Doddapaneni[1] doddapan@bcm.edu

Qingchang Meng[1] qingchang.meng@bcm.edu

Yi Han[1] yhan@bcm.edu

Donna Muzny[1] donnam@bcm.edu

Roger W. Wiseman[2, 3] rwwiseman@wisc.edu

David H. O'Connor[2, 3] dhoconno@wisc.edu

Jeffrey Rogers[1] jr13@bcm.edu

(corresponding author: jr13@bcm.edu; 713-798-7783)

[1]Human Genome Sequencing Center and Department of Molecular and Human Genetics, Baylor College of Medicine, Houston, TX 77030

[2]Wisconsin National Primate Research Center, University of Wisconsin, Madison, WI 53711

[3]Department of Pathology and Laboratory Medicine, University of Wisconsin, Madison, WI 53711

Abstract

Background

The Syrian hamster (*Mesocricetus auratus*) has been suggested as a useful mammalian model for a variety of diseases and infections, including infection with respiratory viruses such as SARS-CoV-2. The MesAur1.0 genome assembly was published in 2013 using whole-genome shotgun sequencing with short-read sequence data. Current more advanced sequencing technologies and assembly methods now permit the generation of near-complete genome assemblies with higher quality and higher continuity.

Findings

Here, we report an improved assembly of the *M. auratus* genome (BCM_Maur_2.0) using Oxford Nanopore Technologies long-read sequencing to produce a chromosome-scale assembly. The total length of the new assembly is 2.46 Gbp, similar to the 2.50 Gbp length of a previous assembly of this genome, MesAur1.0. BCM_Maur_2.0 exhibits significantly improved continuity with a scaffold N50 that is 6.7 times greater than MesAur1.0. Furthermore, 21,616 protein coding genes and 10,459 noncoding genes were annotated in BCM_Maur_2.0 compared to 20,495 protein coding genes and 4,168

noncoding genes in MesAur1.0. This new assembly also improves the unresolved regions as measured by nucleotide ambiguities, where approximately 17.11% of bases in MesAur1.0 were unresolved compared to BCM_Maur_2.0 in which the number of unresolved bases is reduced to 3.00%.

Conclusions

Access to a more complete reference genome with improved accuracy and continuity will facilitate more detailed, comprehensive, and meaningful research results for a wide variety of future studies using Syrian hamsters as models.

Keywords

Syrian hamster, *Mesocricetus auratus*, genome, disease model, COVID-19

Data Description

Introduction

The Syrian hamster (*Mesocricetus auratus*, NCBI:txid10036) has been used in biomedical research for decades because it is a good model for studies of cancer [1] and infectious diseases [2], including SARS-CoV-2, influenza virus, and Ebola virus [3,4]. The use of Syrian hamsters in research has declined [5], likely due to the advancement in genetic and molecular tools for other rodents, especially laboratory mice, and not due to a reduction in the utility of hamsters in biomedical research [2].

Syrian hamsters are particularly important for COVID-19 research. They spontaneously develop more severe lung disease than other animal models, such as wild-type mice, macaques, marmosets, and ferrets [3,6–9]. After intranasal infection, Syrian hamsters consistently show signs of respiratory distress, including laboured breathing, but typically recover after 2 weeks [10]. This is in stark contrast to wild-type laboratory mice that are minimally susceptible to most SARS-CoV-2 strains circulating in 2020, though laboratory mice are more susceptible to certain variants of concern that are circulating in 2021 [11]. Furthermore, a recent analysis has suggested that Syrian hamsters fed a high-fat, high-sugar diet exhibit accelerated weight gain and pathological changes in lipid metabolism, as well as more severe disease outcomes when subsequently infected with SARS-CoV-2 [12]. This result has obvious parallels with observations of the effects of comorbidities in humans suffering from COVID-19.

COVID-19 pathology in Syrian hamsters appears to be due to a dysregulated innate immune response involving signal transducer and activator of transcription factor 2

(STAT2)-dependent type I (IFN-I) and III interferon (IFN-III) signaling [13]. IFN-I signaling can limit virus replication and dissemination and it has been shown that intranasal administration of IFN-I in Syrian hamsters reduces viral load and tissue damage [14]. The human angiotensin-converting enzyme 2 (ACE2) was identified as the cell entry receptor of SARS-CoV-2 [15]. In addition, upon the engagement of ACE2 with SARS-CoV2, cellular transmembrane protease 'serine 2' (TMPRSS2) mediates the priming of viral spike (S) protein by cleaving at S1/S2 site and induces the fusion of viral and host cellular membranes, thus facilitating viral entry into the cells [16]. Human ACE2 and hamster ACE2 receptors had previously been shown to share major sequence homology, which strongly points to interaction with SARS-CoV-2 receptor binding domain (RBD) structures and similar binding affinity [17]. In-silico interaction prediction analysis showed that human and hamster TMPRSS2 are structurally very similar and even with slight differences in amino-acid residue interactions, human and hamster TMPRSS2 activity are identical for residue interactions related to SARS-CoV-2 infectivity [17]. As COVID-19 causes systemic disease in people, precision modeling of specific aspects of pathogenesis will require carefully evaluating similarities and differences in biological processes between humans and Syrian hamsters which, in turn, will require extensive genomic comparisons between the two species.

The currently available reference genome sequence for the Syrian hamster was produced in 2013 using a whole-genome shotgun sequencing approach implementing short read sequencing technology. The resulting MesAur1.0 reference sequence is typical of those produced at that time, containing 237,700 separate contigs with contig N50 of 22,511 bp. The quality and potential of the existing Syrian hamster genome is limited by the

technology that was available at the time of its publication; for example, the cluster of type I Ifn genes was not resolvable with this technology. In this Data Note, we report the production of a new Syrian hamster reference genome that was sequenced using long-read methods on the Oxford Nanopore Technologies (ONT) PromethION platform and assembled into highly contiguous chromosomes using a combination of Flye [18] and Pilon [19] assembly software. The final assembly, BCM_Maur_2.0, improves upon quality and contiguity in comparison with MesAur1.0, with longer contigs and more contiguous sequence, allowing for a more complete reference genome with improved accuracy that will benefit a wide variety of future studies using the Syrian hamster reference genome.

Methods

DNA isolation, library construction, and sequencing

All genomic DNAs for this study were isolated from a single female LVG Golden Syrian hamster (SY011) that was purchased from Charles River, Inc. (Kingston, NY). All procedures were performed in accordance with the guidelines set by the Institutional Animal Care and Use Committee at the University of Wisconsin-Madison. The protocol was approved by the Institutional Animal Care and Use Committee at the University of Wisconsin-Madison (protocol number V00806). Data from this individual are available in NCBI BioProject PRJNA705675, BioSample SAMN18096087. Qiagen AllPrep DNA/RNA Mini kits were used to extract DNA from frozen liver while Qiagen Blood and Cell Culture DNA Midi Kits were used for extractions from frozen kidney. Ultra-high

molecular weight DNA for optical mapping was purified from frozen liver using an Animal Tissue DNA Isolation Kit from Bionano Genomics, Inc. (San Diego, CA).

Oxford Nanopore long-read sequencing

Three Syrian hamster genomic DNA isolates from the same animal were sheared to 10 kb, 20kb and 30kb. The two smaller length fragment libraries were sheared using Covaris gTube and the 30kb targeted size library was fragmented with Diagnode Megarupter 3, all following manufacturer's recommendations. The Oxford Nanopore sequencing libraries were prepared using the ONT 1D sequencing by ligation kit (SQK-LSK109). Briefly, 1-1.5ug of fragmented DNA was repaired with the NEB FFPE repair kit, followed by end repair and A-tailing with the NEB Ultra II end-prep kit. After a clean up step using AMPure beads, the prepared fragments were ligated to ONT specific adapters via the NEB blunt/TA master mix kit. The library underwent a final clean up and was loaded onto a PromethION flow cell per manufacturer's instructions. One library was sequenced per flow cell with standard parameters for 72 hrs. Base-calling was done onboard the PromethION instrument with the use of neuronal network based software (Oxford Nanopore Technologies, UK).

Illumina sequencing

750ng of input DNA was used to generate standard PCR-free Illumina paired-end sequencing libraries. Libraries were prepared using KAPA Hyper PCR-free library reagents (KK8505, KAPA Bio-systems) in Beckman robotic workstations (Biomek FX and FXp models). Total genomic DNA (500 ng) was sheared into fragments of approximately 200-600 bp in a Covaris E220 system (96-well format) followed by purification of the

fragmented DNA using AMPure XP beads. A double size selection step was employed, with different ratios of AMPure XP beads, to select a narrow size band of sheared DNA molecules for library preparation. DNA end-repair and 3'-adenylation were then performed in the same reaction followed by ligation of the barcoded adaptors to create PCR-Free libraries. The resulting libraries were run on the Fragment Analyzer (Advanced Analytical Technologies, Ames, Iowa) to assess library size and presence of remaining adaptor dimers. This was followed by qPCR assay using KAPA Library Quantification Kit and their SYBR FAST qPCR Master Mix to estimate the size and quantify fragment yield.

Sequencing was performed on the NovaSeq 6000 Sequencing System using the S4 reagent kit (300 cycles) to generate 2 x 150 bp paired-end reads. The final concentration of the libraries loaded on flowcells was 400-450 pM. Briefly, the libraries were diluted in an elution buffer and denatured in sodium hydroxide. The denatured libraries were loaded into each lane of the S4 flow cell using the NovaSeq Xp Flow Cell Dock. Each lane included ~1% of a PhiX control library for run quality control.

Genome Assembly

We generated 221 Gbp of sequence data using the ONT PromethION platform (NCBI BioSample SAMN18096087) representing 88X coverage of the expected 2.5 Gbp Syrian hamster genome. The sequencing reads have an N50 of 15,730 bp. The Flye assembler v2.8.1 [18] was used to generate the initial *de novo* genome assembly. Pilon uses high quality Illumina reads mapped to an assembly to correct errors arising from the lower quality ONT sequencing data. Pilon correction using 30X of Illumina data from the same individual was performed. Next, we applied Bionano optical mapping data to the assembly

which links contigs into scaffolds approaching chromosome scale. Based on the Bionano Molecule Quality Report (MQR), the optical map N50 (>=150 kbp and minSites >= 9) was 0.2341 Mbp and the average label density (>= 150 kbp) was 17.40/100 kbp. This gave an effective molecule coverage of 125.38X. Bionano made 84 conflict cuts to the Flye assembly. The completed assembly has been submitted to NCBI under accession GCA_017639785.1. NCBI performed gene annotation using RNA-Seq data from multiple tissues including lung, trachea, brain, olfactory bulb and small intestine that are targets for SARS-CoV-2 infection (NCBI BioProject PRJNA675865).

Quality assessment

To assess the quality of our assembly compared to the previous MesAur1.0 we used Quast v5.0.2 [20] together with MUMmer v3.23 [21] and Assemblytics [22]. These tools provided a detailed comparison between these assemblies. In addition, the Illumina reads from the original reference (NCBI SRA SRR413408) were mapped to our assembly and the MesAur1.0 reference using BWA v0.7.17 [23] and Quast was used to obtain discordant pair statistics.

The software Benchmarking Universal Single-Copy Orthologs (BUSCO) v3.0.2 [24] was used for quality assessment of the genome assembly. BUSCO is based on the concept that single-copy orthologs should be highly conserved among closely related species. BUSCO performs gene annotation on an assembly and reports the number of gene models generated. BUSCO was performed using the OrthoDB v9 (odb9) release consisting of 6192 genes shared across the superorder Euarchontoglires [25] of which the Syrian hamster is a member.

Results

The initial Flye assembly consisted of 2.38 Gbp of sequence across 6,741 scaffolds with a scaffold N50 of 10.56 Mbp (**Table 1**). Pilon polishing of the Flye assembly had little effect on these metrics but significant improvements were obtained when Bionano optical mapping results were incorporated in the assembly. As shown in **Table 1**, this step reduced the total number of scaffolds in the final assembly by 395 (5.9%) while increasing the N50 scaffold length by more than 8-fold to 85.18 Mbp.

Table 1. Assembly statistics for BCM_Maur_2.0 versus the MesAur1.0 Syrian hamster assembly

Parameter	MesAur1.0	Flye	Flye + Pilon	Flye + Pilon + Bionano (BCM_Maur_2.0)
Assembly length (bp)	2,504,908,775	2,381,258,546	2,383,228,608	2,457,062,007
Ungapped length (bp)	2,076,159,990	2,381,254,546	2,383,226,373	2,383,228,883
Number of scaffolds	21,483	6,741	6,741	6,346
N50 scaffold length (bp)	12,753,307	10,564,357	10,573,641	85,184,847
Number of contigs	237,699	6,781	6,779	7,057
N50 contig length (bp)	22,512	10,022,145	10,097,207	9,471,653

Of the 6192 BUSCO gene models, 88.91% were annotated as complete genes in the initial Flye assembly (**Table 2**). Pilon polishing of this Flye-alone assembly added another 295 genes that were annotated completely and increased this proportion up to 93.67% of the BUSCO gene model dataset. Improvements in assembly scaffolding resulting from the Bionano optical mapping step together with Pilon error correction decreased the proportions of fragmented and missing BUSCO gene models in the new assembly to 2.9% and 3.2% respectively, also improvements over the MesAur1.0 assembly. This advance translates to an additional 305 complete BUSCO genes identified in the new assembly.

Table 2. BUSCO statistics for BCM_Maur_2.0 versus the MesAur1.0 Syrian hamster assembly

	MesAur1.0	Flye	Flye + Pilon	Flye + Pilon + Bionano (BCM_Maur_2.0)
Complete[a]	88.97%	88.91%	93.67%	93.90%
Complete and single-copy	88.47%	88.05%	92.80%	93.12%
Complete and duplicated	0.50%	0.86%	0.87%	0.78%
Fragmented	5.89%	5.99%	3.04%	2.87%
Missing	5.14%	5.10%	3.29%	3.23%

[a]6192 gene models were included in this analysis

Assembly Comparisons

We also performed other comparisons between the two assemblies. The total length of the BCM_Maur_2.0 assembly is 2.46 Gbp compared to the previous version's 2.50 Gbp. Despite having a similar total length, BCM_Maur_2.0 shows an improved continuity with a scaffold N50 that is 6.7 times higher than MesAur1.0 (**Table 1**); the L50 (i.e. the number of contigs longer than or equal to the N50 length) of BCM_Maur_2.0 is 22 compared to MesAur1.0's 121. The longest scaffold of BCM_Maur_2.0 (187 Mb) is 2.35 times larger than the longest scaffold from the previous assembly. N50 is calculated in the context of the assembly size rather than the genome size, so the NG50 statistic was used to directly compare the different assemblies. NG50 is the same as N50 except that it reports the length of the contig at which the size-ordered contigs (longest to shortest) collectively reaches 50% of the known or estimated genome size [26]. **Figure 1** illustrates the additional contig sequence length generated from the BCM_Maur_2.0 assembly when compared to MesAur1.0 at the same NG50. The BCM_Maur_2.0 assembly further improves the unresolved regions as measured by nucleotide ambiguities (i.e. number of N's). Approximately 17.11% of bases in MesAur1.0 were unresolved. BCM_Maur_2.0 reduces the number of unresolved bases to 3.00%, with only very small gaps throughout the entire genome. **Figure 2** displays the overall increase in continuity of the BCM_Maur_2.0 assembly with longer contigs than the MesAur1.0 assembly and fewer short contigs.

To establish the correctness of the structure and completeness of BCM_Maur_2.0, we also leveraged the Illumina short-reads that were published as part of the MesAur1.0

assembly project. When mapping the MesAur1.0 Illumina reads back to the MesAur1.0 reference, only 92.19% reads mapped successfully. When the same Illumina reads were instead mapped to BCM_Maur_2.0, 97.32% mapped successfully. When considering only properly paired reads, 75.76% and 87.62% mapped to MesAur1.0 and BCM_Maur_2.0, respectively.

Alignments between the current and previous Syrian hamster assemblies performed by NCBI [27] show that BCM_Maur_2.0 covers 98.95% of MesAur1.0 while MesAur1.0 only covers 86.67% of BCM_Maur_2.0. This together with the additional 307 Mbp of ungapped sequence in BCM_Maur_2.0 indicates that BCM_Maur_2.0 is a more complete representation of the Syrian hamster genome. The percent identity in the regions aligned between the two assemblies is 99.76%.

Transcript and Protein Alignments and Annotation Comparisons

NCBI annotation of BCM_Maur_2.0 [27] with Syrian hamster transcript and protein data show this assembly to be of high quality. Transcript alignments of Syrian hamster RefSeq (n=273), Genbank (n=751), and EST (n=558) data to BCM_Maur_2.0 show 99.44% or more average percent identity and 98.88% or more average percent coverage. Alignments of these same transcript datasets to MesAur1.0 show 99.13% or more average percent identity and 93.49% or more average percent coverage. Protein alignments of Syrian hamster RefSeq (n=261) and Genbank (n=485) data to BCM_Maur_2.0 show 80.95% or more average percent identity and 89.18% or more average percent coverage. Alignments of these same protein datasets to MesAur1.0

show 80.57% or more average percent identity and 84.87% or more average percent coverage.

NCBI annotated 21,616 protein coding genes and 10,459 noncoding genes in BCM_Maur_2.0 compared to 20,495 protein coding genes and 4,168 noncoding genes in MesAur1.0 [28]. Only 7% of gene annotations are identical between BCM_Maur_2.0 and MesAur1.0, suggesting that a number of previous errors have been corrected, though some differences are likely to be real differences between the animals used for the different assemblies. Minor changes between BCM_Maur_2.0 and MesAur1.0 were made in 46% of gene annotations and major changes were made in 15% of gene annotations.

Interferon type 1 alpha gene cluster

Given the importance of type I interferon responses during SARS-CoV-2 infection, we next compared the interferon type I alpha gene cluster in the BCM_Maur_2.0 assembly relative to this genomic region in the original MesAur1.0 assembly. The MesAur1.0 scaffold NW_004801649.1 includes annotations for four interferon type I alpha loci but this genomic sequence is riddled with numerous gaps. Of these four candidate loci, only LOC101824534 appears to contain a complete interferon alpha-12-like coding sequence with the ability to encode a predicted protein of 183 amino acids (XP_005074343.1). The LOC101824794 gene sequence can only encode a 162 amino acid protein due to a 5' truncation. The remaining pair of candidate genes (LOC101836618 and LOC101836898) appear to have aberrant transcript models that have fused putative exons from neighboring loci. In mice and humans, the interferon alpha gene cluster is flanked by

single copy interferon beta 1 (Ifnb1) and interferon epsilon (Ifne) genes. Although neither of these genes are present on the MesAur1.0 scaffold NW_004801649.1, this assembly does contain a Ifne gene on a short 2,408 bp contig that is predicted to code for a protein of 192 amino acids. These observations emphasize the need for an improved genomic assembly for Syrian hamsters given that the interferon alpha gene cluster includes more than a dozen tightly linked functional genes plus multiple pseudogenes in a wide variety of species including mice and humans.

The interferon type I alpha gene cluster is contained on the NW_024429197.1 super scaffold that spans nearly 75 Mbp in the BCM_Maur_2.0 assembly. **Figure 3** illustrates this genomic region in comparison with the well-studied interferon type I alpha gene cluster of C57BL/6J mice (NC_000070.7). Fourteen predicted interferon type I alpha genes as well as five pseudogenes lie within a span of 196 Kbp of the new Syrian hamster assembly (**Figure 3** and **Supplemental Table 1**). This genomic organization is quite comparable to that observed in the mouse genome where there are also fourteen functional interferon alpha genes and four pseudogenes. This hamster gene cluster is flanked by Ifnb1 and Ifne genes consistent with expectations from the mouse and other species. The NCBI annotations characterize twelve of these genes as interferon alpha-12-like with predicted coding sequences of 190 amino acids (**Supplemental Table 1**). The remaining pair of functional genes (LOC101824794 and LOC121144100) are listed as interferon alpha-9-like and they encode shorter predicted proteins of 178 and 182 amino acids, respectively. The increased length of this genomic region in the mouse assembly is largely due to the presence of the interferon zeta gene family (Ifnz, Gm13271, Gm13272, etc.). This Ifnz gene family appears to be absent in Syrian hamsters since the

closest matches to predicted hamster protein sequences are only 28% identical. The availability of a contiguous hamster genomic sequence and associated transcriptional regulatory elements for this complex immune gene region may be helpful for investigators who are interested in unravelling mechanisms that control interferon expression during infections with SARS-CoV-2 as well as challenges with other viral pathogens.

Conclusion

The improved Syrian hamster assembly and annotation described here will facilitate research into this important animal model for COVID-19. Specifically, reagents for studying immune responses in hamsters have lagged behind those available for laboratory mice. BCM_Maur_2.0 will facilitate the identification of cross-reactive reagents originally developed to study immunity in other species. Additionally, a more accurate genome assembly will improve the analyses of host responses to infection by enabling more accurate interpretation of RNA-seq experiments.

Relative to other recent assemblies that use a combination of long-read sequencing and short-read polishing, this genome assembly and annotation compares very favorably. The scaffold N50 of >85 Mbp is quite consistent with other long read assemblies. The contig N50 and total number of scaffolds or contigs are likewise reasonable and consistent with other similar reference genomes. The number of protein coding genes identified is within the expected range, although additional attention will likely be needed to resolve duplicated, repetitive gene loci, potentially leveraging recent advances in ultralong read sequencing.

What additional genomic resources would be needed to make hamsters a better model for COVID-19? Deep long read transcriptome analysis of multiple tissues and ages would be the best next step, in order to define not just the genes expressed but the alternative splicing of genes across tissues and developmental stages. Also, long read RNA-seq of tissues following experimental challenge with SARS-CoV-2 and other viruses would facilitate improvements in the quality of antiviral gene models.

The availability of higher accuracy sequences should lead to the development of specific reagents for monitoring immune responses. For example, epitopes that are shared between hamsters and other rodents can be used to identify monoclonal antibody reagents for flow cytometry that are predicted to be cross-reactive. Additional reagent development will be enabled by creating synthetic versions of hamster proteins that can be used as immunogens to make hamster-specific antibodies.

One surprising motivation for this study is that Syrian hamsters, which were quickly identified as a high value model for COVID-19, did not have a higher quality reference genome at the start of the pandemic. While we worked quickly to generate this data and make it available to the scientific community, better preparedness will be critical for future unexpected epidemics. To this end, we would encourage investment in continued refinement and improvement of reference genomes for all of the rodent and nonhuman primate models that are commonly used to study viruses in order to prevent this situation from recurring in the future. Such an investment would also yield improved genomic resources that would provide broad benefit to the entire scientific community.

Availability of Supporting Data and Materials

The MesAur1.0 genome assembly is available in the NCBI database under BioProject PRJNA77669 (GenBank accession GCA_000349665.1). The new BCM_Maur_2.0 genome assembly is available in the NCBI data repository under BioProject PRJNA705675 (GenBank accession GCA_017639785.1). The submission of the Oxford Nanopore long read and Illumina short read DNA sequence data is in progress and will be available through the NCBI data archive soon. The PacBio RNA-Seq data from multiple tissues including lung, trachea, brain, olfactory bulb and small intestine are available under NCBI BioProject PRJNA675865.

Additional Files

Supplementary Table 1. Predicted genes in the Interferon type 1 alpha cluster of the BCM_Maur_2.0 assembly.

Abbreviations

ACE2: angiotensin-converting enzyme 2; BCM: Baylor College of Medicine; bp: base pairs; BUSCO: Benchmarking Universal Single-Copy Orthologs; BWA: Burrows-Wheeler Aligner; COVID-19: coronavirus disease 2019; EST: expressed sequence tag; FFPE: formalin-fixed, paraffin-embedded; Gbp: gigabase pairs; GC: guanine-cytosine; IFN: interferon; kbp: kilobase pairs; Mbp: megabase pairs; MQR: Molecule Quality Report; NCBI: National Center for Biotechnology Information; NEB: New England BioLabs; ng: nanogram; ONT: Oxford Nanopore Technologies; PCR: polymerase chain reaction; RBD:

receptor-binding domain; RNA-Seq: RNA-sequencing; SARS-CoV-2: severe acute respiratory syndrome coronavirus 2; STAT2: signal transducer and activator of transcription factor 2; TMPRSS2: transmembrane protease serine 2

Competing interests

The authors declare that they have no competing interests.

Funding

This research was supported by contract HHSN272201600007C awarded to DHO from the National Institute of Allergy and Infectious Diseases of the National Institutes of Health. The content of this publication is solely the responsibility of the authors and does not necessarily represent the official views of the National Institutes of Health.

Authors' Contributions

R.A.H. performed genome assembly and quality assessment, data and metadata submission, and contributed to manuscript preparation. F.S. and M.M. performed assembly assessment and comparison analyses. T.M.P. and R.W.W. performed transcript and annotation comparisons. D.H.O. managed experimental design and oversight and coordinated manuscript preparation. H.D., Q.M. and Y.H. developed, optimized and implemented protocols for ONT PromethION sequencing. M.R., D.M., J.A.K. and J.R. performed project and/or data management. R.A.H., D.H.O., D.T.L., T.M.P., R.W.W., M.M., F.S. and J.R. wrote the manuscript. All authors approved the manuscript.

Acknowledgements

We are extremely grateful to Dr. Tadashi Maemura for collecting the Syrian hamster tissues that were used for the sequence analyses described here. We also thank Dr. Benjamin tenOever for sharing Syrian hamster RNA-Seq datasets generated by his group prior to publication.

Figure 1: Cumulative length and continuity comparison of MesAur1.0 and BCM_Maur_2.0. This summarizes the length of contigs/scaffolds across the assemblies. Given the length of contigs, the NG50 (mid x-axis) summarizes the sequence length of the shortest contig/scaffold at 50% of the total genome length. For genome length, the established MesAur1.0 was used.

Figure 2: Contig length and count comparison between BCM_Maur_2.0 and MesAur1.0. Log length of contigs on the X axis and normalized count on the Y axis comparing BCM_Maur_2.0 assembly and the previous assembly. Contigs from BCM_Maur_2.0 are shown red and contigs for MesAur1.0 are shown in gray.

Figure 3: Comparison of interferon type 1 alpha gene cluster between BCM_Maur_2.0 and GCRm39 mouse genome assembly. The genomic intervals illustrated here are defined by the flanking interferon beta 1 and interferon epsilon genes in both species. Accession numbers for each genomic sequence are indicated on the right with genomic coordinates for the extracted intervals shown below their respective accession numbers. Predicted interferon type 1 alpha genes are highlighted in blue while pseudogenes are depicted with open symbols and labelled below each assembly.

References

1. LaRocca CJ, Han J, Gavrikova T, Armstrong L, Oliveira AR, Shanley R, et al.. Oncolytic adenovirus expressing interferon alpha in a syngeneic Syrian hamster model for the treatment of pancreatic cancer. *Surgery*. 2015; doi: 10.1016/j.surg.2015.01.006.

2. McCann KE, Sinkiewicz DM, Norvelle A, Huhman KL. De novo assembly, annotation, and characterization of the whole brain transcriptome of male and female Syrian hamsters. *Sci Rep*. 2017; doi: 10.1038/srep40472.

3. Chan JF-W, Zhang AJ, Yuan S, Poon VK-M, Chan CC-S, Lee AC-Y, et al.. Simulation of the Clinical and Pathological Manifestations of Coronavirus Disease 2019 (COVID-19) in a Golden Syrian Hamster Model: Implications for Disease Pathogenesis and Transmissibility. *Clin Infect Dis*. 2020; doi: 10.1093/cid/ciaa325.

4. Prescott J, Falzarano D, Feldmann H. Natural Immunity to Ebola Virus in the Syrian Hamster Requires Antibody Responses. *J Infect Dis*. 2015; doi: 10.1093/infdis/jiv203.

5. Gao M, Zhang B, Liu J, Guo X, Li H, Wang T, et al.. Generation of transgenic golden Syrian hamsters. *Cell Res*. 2014; doi: 10.1038/cr.2014.2.

6. Imai M, Iwatsuki-Horimoto K, Hatta M, Loeber S, Halfmann PJ, Nakajima N, et al.. Syrian hamsters as a small animal model for SARS-CoV-2 infection and countermeasure development. *Proc Natl Acad Sci U S A*. 2020; doi: 10.1073/pnas.2009799117.

7. Rockx B, Kuiken T, Herfst S, Bestebroer T, Lamers MM, Oude Munnink BB, et al.. Comparative pathogenesis of COVID-19, MERS, and SARS in a nonhuman primate model. *Science*. 2020; doi: 10.1126/science.abb7314.

8. Rogers TF, Zhao F, Huang D, Beutler N, Burns A, He W-T, et al.. Isolation of potent SARS-CoV-2 neutralizing antibodies and protection from disease in a small animal model. *Science*. 2020; doi: 10.1126/science.abc7520.

9. Shi J, Wen Z, Zhong G, Yang H, Wang C, Huang B, et al.. Susceptibility of ferrets, cats, dogs, and other domesticated animals to SARS-coronavirus 2. *Science*. 2020; doi: 10.1126/science.abb7015.

10. Muñoz-Fontela C, Dowling WE, Funnell SGP, Gsell P-S, Riveros-Balta AX, Albrecht RA, et al.. Animal models for COVID-19. *Nature*. 2020; doi: 10.1038/s41586-020-2787-6.

11. Montagutelli X, Prot M, Levillayer L, Salazar EB, Jouvion G, Conquet L, et al.. The B1.351 and P.1 variants extend SARS-CoV-2 host range to mice. bioRxiv.

12. Port JR, Adney DR, Schwarz B, Schulz JE, Sturdevant DE, Smith BJ, et al.. Western diet increases COVID-19 disease severity in the Syrian hamster. *bioRxiv*. 2021; doi: 10.1101/2021.06.17.448814.

13. Boudewijns R, Thibaut HJ, Kaptein SJF, Li R, Vergote V, Seldeslachts L, et al.. STAT2

signaling restricts viral dissemination but drives severe pneumonia in SARS-CoV-2 infected hamsters. *Nat Commun*. 2020; doi: 10.1038/s41467-020-19684-y.

14. Hoagland DA, Møller R, Uhl SA, Oishi K, Frere J, Golynker I, et al.. Leveraging the antiviral type I interferon system as a first line of defense against SARS-CoV-2 pathogenicity. *Immunity*. 2021; doi: 10.1016/j.immuni.2021.01.017.

15. Brooke GN, Prischi F. Structural and functional modelling of SARS-CoV-2 entry in animal models. *Sci Rep*. 2020; doi: 10.1038/s41598-020-72528-z.

16. Hoffmann M, Kleine-Weber H, Schroeder S, Krüger N, Herrler T, Erichsen S, et al.. SARS-CoV-2 Cell Entry Depends on ACE2 and TMPRSS2 and Is Blocked by a Clinically Proven Protease Inhibitor. *Cell*. 2020; doi: 10.1016/j.cell.2020.02.052.

17. Rizvi ZA, Dalal R, Sadhu S, Kumar Y, Shrivastava T, Gupta SK, et al.. Immunological and cardio-vascular pathologies associated with SARS-CoV-2 infection in golden syrian hamster. Cold Spring Harbor Laboratory.

18. Kolmogorov M, Yuan J, Lin Y, Pevzner PA. Assembly of long, error-prone reads using repeat graphs. *Nat Biotechnol*. 2019; doi: 10.1038/s41587-019-0072-8.

19. Walker BJ, Abeel T, Shea T, Priest M, Abouelliel A, Sakthikumar S, et al.. Pilon: an integrated tool for comprehensive microbial variant detection and genome assembly improvement. *PLoS One*. 2014; doi: 10.1371/journal.pone.0112963.

20. Gurevich A, Saveliev V, Vyahhi N, Tesler G. QUAST: quality assessment tool for genome assemblies. *Bioinformatics*. 2013; doi: 10.1093/bioinformatics/btt086.

21. Kurtz S, Phillippy A, Delcher AL, Smoot M, Shumway M, Antonescu C, et al.. Versatile and open software for comparing large genomes. *Genome Biol*. 2004; doi: 10.1186/gb-2004-5-2-r12.

22. Nattestad M, Schatz MC. Assemblytics: a web analytics tool for the detection of variants from an assembly. *Bioinformatics*. 2016; doi: 10.1093/bioinformatics/btw369.

23. Li H. Aligning sequence reads, clone sequences and assembly contigs with BWA-MEM. arXiv [q-bio.GN].

24. Seppey M, Manni M, Zdobnov EM. BUSCO: Assessing Genome Assembly and Annotation Completeness. *Methods Mol Biol*. 2019; doi: 10.1007/978-1-4939-9173-0_14.

25. : BUSCO. https://busco-archive.ezlab.org/v3/datasets/euarchontoglires_odb9.tar.gz Accessed 2021 Jun 4.

26. Alhakami H, Mirebrahim H, Lonardi S. A comparative evaluation of genome assembly reconciliation tools. *Genome Biol*. 2017; doi: 10.1186/s13059-017-1213-3.

27. : Mesocricetus auratus Annotation Report. https://www.ncbi.nlm.nih.gov/genome/annotation_euk/Mesocricetus_auratus/103/ Accessed 2021 Jun 4.

28. : Mesocricetus auratus Annotation Report. https://www.ncbi.nlm.nih.gov/genome/annotation_euk/Mesocricetus_auratus/102/ Accessed 2021 Jun 4.

Figure 1

Click here to access/download;Figure;Figure1.pdf

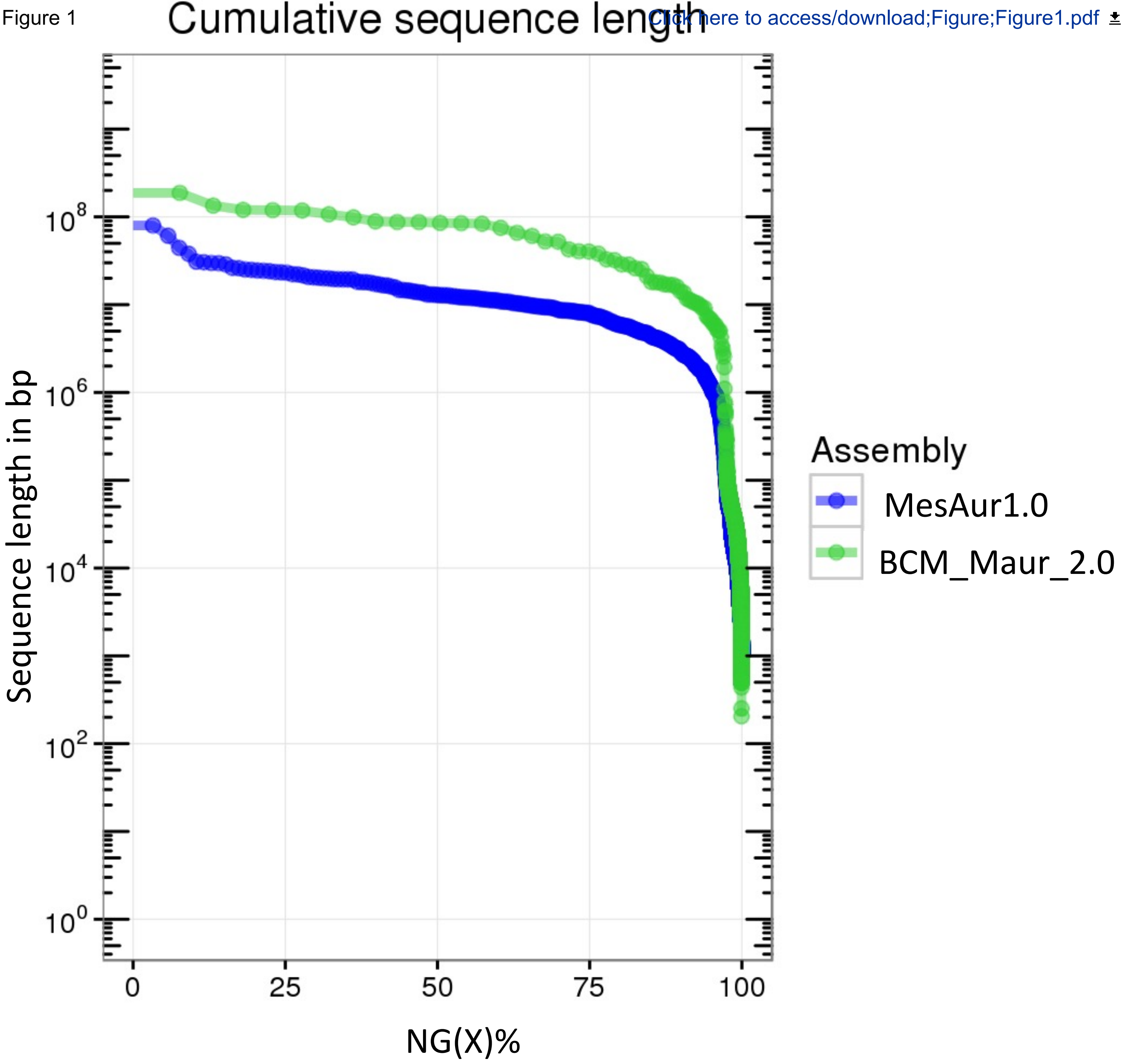

Figure 2

Click here to access/download;Figure;Figure2.pdf

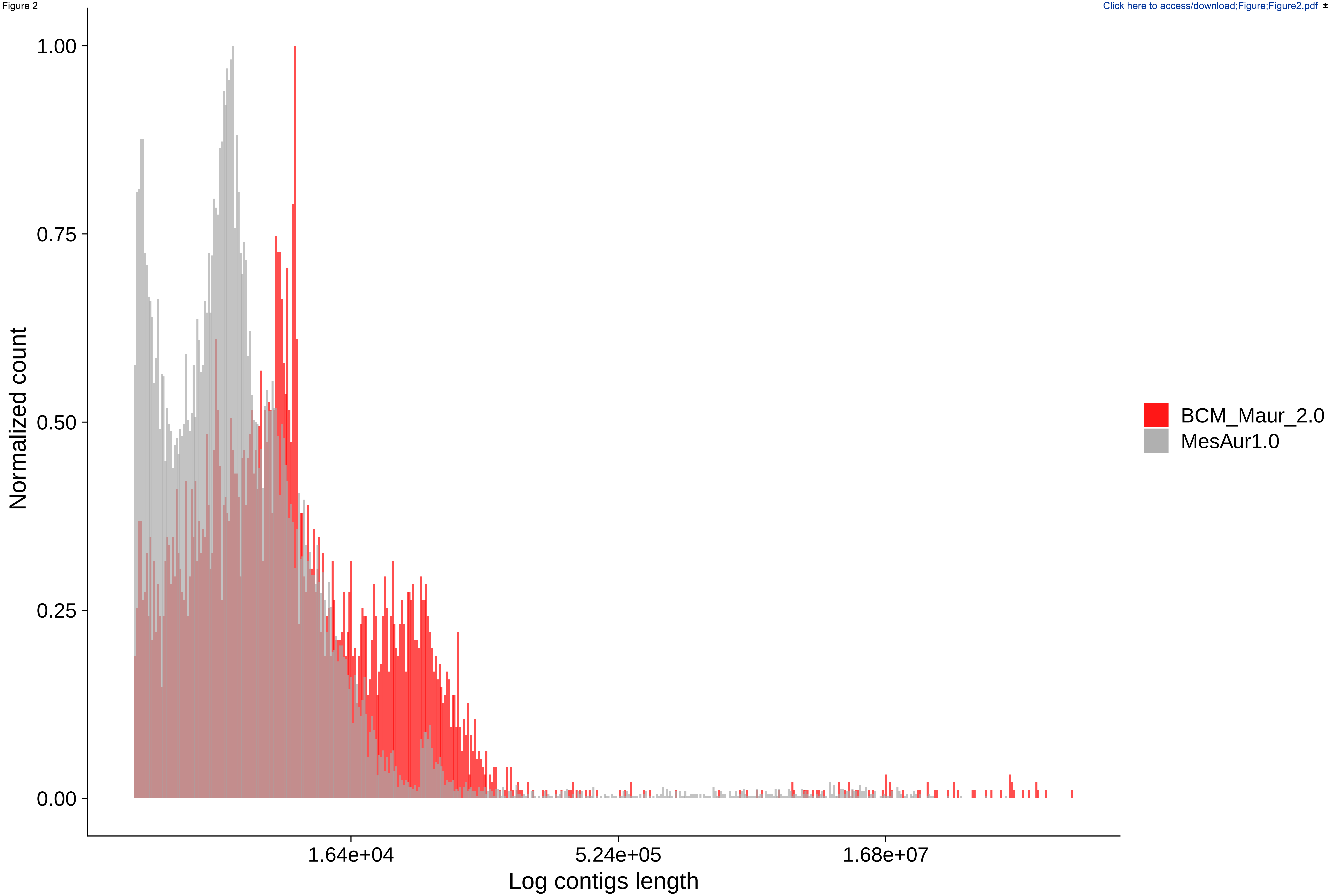

Figure 3

Click here to access/download;Figure;Figure3.pdf

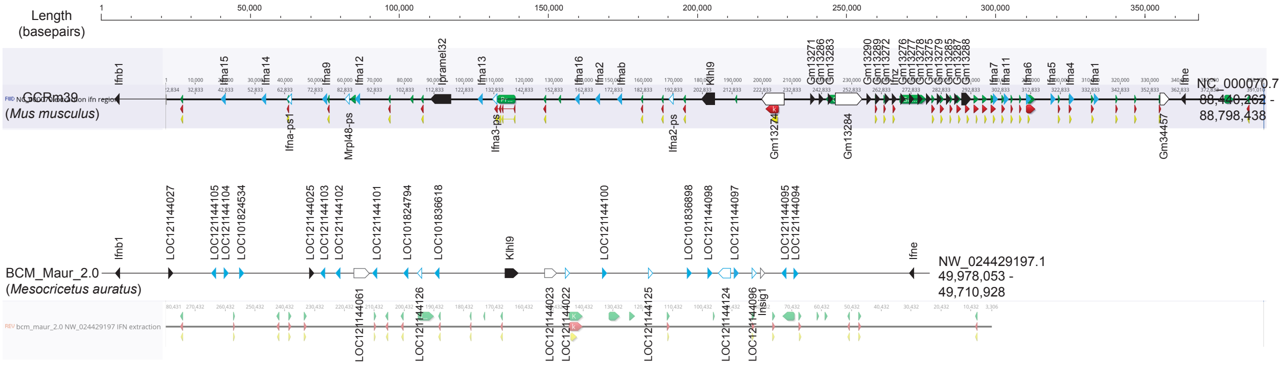

Click here to access/download
Supplementary Material
Supplemental Table 1. Interferon alpha genes.xlsx

Personal Cover
Click here to access/download;Personal Cover;hamster cover letter 070221.pdf

July 2, 2021

Baylor College of Medicine
HUMAN GENOME
SEQUENCING CENTER
ONE BAYLOR PLAZA
ALKEK BUILDING, 15th FLOOR
MS: BCM226
HOUSTON, TEXAS 77030
713-798-6539
713-798-5741 FAX

Scott Edmunds, PhD
Editor-in-Chief
GigaScience

Dear Dr. Edmunds,

We are pleased to submit our manuscript "Construction of a new chromosome-scale, long-read reference genome assembly of the Syrian hamster, *Mesocricetus auratus*" for consideration for publication by *GigaScience*.

This new reference genome assembly constitutes an important research resource that will be valuable to a wide variety of investigators. Most significantly, Syrian hamsters are uniquely suited as models for COVID-19 research due to their spontaneous development of more severe lung disease compared to other animal models. COVID-19 pathology in Syrian hamsters very closely mirrors pathology in humans so extensive genomic comparison is required to evaluate similarities and differences in their biological processes. The previous reference genome assembly for *Mesocricetus auratus* was produced using short-read sequencing technology that was state-of-the-art at the time of its publication, but is now unsatisfactory due to various limitations. Current more advanced sequencing technologies and assembly methods now permit the generation of near-complete genome assemblies with higher quality and higher continuity. Our manuscript details the generation of an improved *Mesocricetus auratus* reference genome implementing long-read sequencing technologies that result in a more contiguous and complete reference genome. We also describe specific examples of COVID-19 related genes that were not fully resolved in the previous assembly but are now resolved in the new BCM_Maur_2.0 assembly.

Long-read sequencing technology from the Oxford Nanopore Technologies (ONT) PromethION platform was used to generate 221 Gbp of sequence data (BioSample SAMN18096087). Initial *de novo* assembly was performed with the Flye assembler and low-quality ONT sequence data was error-corrected with Illumina sequence data using Pilon software. The completed assembly is available in NCBI under accession (GCA_017639785.1). Pacific Biosciences (PacBio) Iso-Seq (SRR12589345) and RNA-Seq (PRJNA675865) data used for gene annotation are also available in NCBI. All the data we used in this project, as well as the final assembly itself, has been submitted to or is already available through public databases.

GigaScience has published many new and updated genome assembly announcements, providing ample precedent for consideration of this manuscript by the journal. To our knowledge, a new *Mesocricetus auratus* reference genome has not been produced anywhere else. The authors have no competing interests and the content of this manuscript has not been published, or submitted for publication elsewhere. All authors approve the manuscript for submission.

We respectfully suggest the following investigators with experience in genomics and genome assemblies, COVID-19 models and/or transcriptomic analysis of this species as potential reviewers for this manuscript:

Florian Krammer, Ph.D.
Dept. of Microbiology
Icahn School of Medicine at Mount Sinai
New York, New York
florian.krammer@mssm.edu

Wesley Warren, Ph.D.
Professor of Genomics
Bond Life Sciences Center
University of Missouri
Columbia, Missouri
warrenwc@missouri.edu

Christopher Mason, Ph.D.
Institute for Computational Biomedicine
Weill Cornell Medical School
New York, New York
Chm2042@med.cornell.edu

Kim Huhman, Ph.D.
Neuroscience Institute
Georgia State University
Atlanta, GA
khuhman@gsu.edu

We thank you in advance for your consideration.

Sincerely,

Jeffrey A Rogers

Associate Professor
Human Genome Sequencing Center and
Dept. of Molecular and Human Genetics
