# Supplementary material for: Construction of a new chromosome-scale, long-read reference genome assembly for the Syrian hamster, Mesocricetus auratus
Source: Gigascience. 2022 May 28;11:giac039. doi: 10.1093/gigascience/giac039 (PMC9155146; doi:10.1093/gigascience/giac039)
Supplement: giac039_GIGA-D-21-00197_Revision_1 [file giac039_giga-d-21-00197_revision_1.pdf]

GigaScience

Construction of a new chromosome-scale, long-read reference genome assembly for the Syrian hamster, Mesocricetus auratus

--Manuscript Draft--

**Manuscript Number:**	GIGA-D-21-00197R1
**Full Title:**	Construction of a new chromosome-scale, long-read reference genome assembly for the Syrian hamster, Mesocricetus auratus
**Article Type:**	Data Note
**Funding Information:**	division of intramural research, national institute of allergy and infectious diseases (HHSN272201600007C) — Dr David H. O'Connor
**Abstract:**	Background<br>The Syrian hamster (Mesocricetus auratus) has been suggested as a useful mammalian model for a variety of diseases and infections, including infection with respiratory viruses such as SARS-CoV-2. The MesAur1.0 genome assembly was generated in 2013 using whole-genome shotgun sequencing with short-read sequence data. Current more advanced sequencing technologies and assembly methods now permit the generation of near-complete genome assemblies with higher quality and greater continuity.<br>Findings<br>Here, we report an improved assembly of the M. auratus genome (BCM_Maur_2.0) using Oxford Nanopore Technologies long-read sequencing to produce a chromosome-scale assembly. The total length of the new assembly is 2.46 Gbp, similar to the 2.50 Gbp length of a previous assembly of this genome, MesAur1.0. BCM_Maur_2.0 exhibits significantly improved continuity with a scaffold N50 that is 6.7 times greater than MesAur1.0. Furthermore, 21,616 protein coding genes and 10,459 noncoding genes are annotated in BCM_Maur_2.0 compared to 20,495 protein coding genes and 4,168 noncoding genes in MesAur1.0. This new assembly also improves the unresolved regions as measured by nucleotide ambiguities, where approximately 17.11% of bases in MesAur1.0 were unresolved compared to BCM_Maur_2.0 in which the number of unresolved bases is reduced to 3.00%.<br>Conclusions<br>Access to a more complete reference genome with improved accuracy and continuity will facilitate more detailed, comprehensive, and meaningful research results for a wide variety of future studies using Syrian hamsters as models.
**Corresponding Author:**	Jeffrey Rogers, Ph.D.<br>Baylor College of Medicine<br>Houston, Texas UNITED STATES
**Corresponding Author Secondary Information:**	
**Corresponding Author's Institution:**	Baylor College of Medicine
**Corresponding Author's Secondary Institution:**	
**First Author:**	R. Alan Harris
**First Author Secondary Information:**	
**Order of Authors:**	R. Alan Harris
	Muthuswamy Raveendran, Ph.D.,
	Dustin T Lyfoung
	Fritz J Sedlazeck
	Medhat Mahmoud
	Trent M Prall

Powered by Editorial Manager® and ProduXion Manager® from Aries Systems Corporation

	Julie A Karl
	Harshavardhan Doddapaneni
	Qingchang Meng
	Yi Han
	Donna Muzny
	Roger W Wiseman
	David H. O'Connor
	Jeffrey Rogers
**Order of Authors Secondary Information:**	
**Response to Reviewers:**	GIGA-D-21-00197<br>Harris et al.<br>Title: Construction of a new chromosome-scale, long read reference genome assembly for the Syrian hamster, Mesocricetus auratus<br><br>Response to Reviewer Critiques<br><br>We thank both reviewers for their largely positive evaluations of our manuscript. We have now addressed all the specific issues identified in their critiques, as outlined below. The reviewer comments are presented in Arial font and our responses are in Times New Roman font.<br><br>Reviewer #1<br><br>Summary: In this manuscript, Harris et al. detail the methods they used to create a new reference genome for the Syrian hamster, which is an important model for respiratory disease pathogens. They used several different sequencing technologies to generate the contigs and scaffolds for their new assembly, and achieved a relatively continuous end product. The analysis is suitable for the "genome report" style format (with one omission detailed below in my comments); however, the manuscript suffers from some awkward phrasing and grammar errors in the results and methods. I list my comments below in the relative order in which I encountered them in the manuscript. Since the authors did not provide line numbers in their submission, I provide my comments as a block listing of questions/suggestions/critiques.<br><br>We thank Reviewer #1 for their overall positive assessment of our manuscript.<br><br>Section titled "oxford nanopore long-read sequencing": The description of the shearing is awkward. I recommend revising the first sentence to state that the genomic DNA isolates were sheared to three lengths (without providing these lengths in the sentence). In subsequent sentences, provide the lengths in situ with the methods used to prepare them. Also, it is unclear why three different fragment lengths were used here for oxford nanopore sequencing. Given that these fragment lengths are relatively similar in size (e.g. not disparate lengths similar to recent ultra-long nanopore read preps of >100kb), it would be very helpful to the reader if justification was given for this approach.<br><br>We thank the reviewer for pointing out these issues with the text. We have now re-written this paragraph (lines 121 - 126) to increase clarity and have added the recommended explanations.<br><br>Section titled "Genome assembly": This entire paragraph is awkwardly phrased with numerous past- or present-tense changes. Additionally, the reference to the Pilon polisher needs to be cited, and details need to be provided on what settings were used for Pilon polishing (it is often recommended to correct only indels and to omit gap-filling) and how many iterations of polishing were used. Details are missing on how BioNano optical maps were generated, and what DNA was used as input in the process. Also, what software was used to compare BioNano optical maps, and with what settings? Finally, it appears that the RNA-seq data used by NCBI for annotation

Powered by Editorial Manager® and ProduXion Manager® from Aries Systems Corporation

was used in another study. Citation to that study would be required so that the reader is aware that the data resulted from different individuals other than the reference individual sequenced in this analysis.

The section titled “Genome assembly” has now been extensively re-written (lines 158 – 168). We have added the citation to Pilon software and included more details on the analyses using Pilon (lines 164 – 168). We have also incorporated significantly more information about the methods used in the optical mapping analyses (lines 170 – 205) and have included the citation to the publication that reported the RNA-seq studies that provided the transcriptome data for annotation of this genome (line 209).

Section titled "Assembly Comparisons": What is the expected c-value of the Syrian Hamster genome? Also, what is the karyotype count? Are any of the chromosomes metacentric or acrocentric? Were any satellite regions identified and annotated in this assembly? Finally, I would have preferred that assembly comparisons be conducted with feature response curves, such as those produced by the program "FRC_align" as this provides a useful metric to assess assembly "correctness" by length.

Despite substantial effort, we cannot find information about the c-value for M. auratus. However, in lieu of c-value data, we now provide the results of k-mer analysis of genome length using both SGA preqc and Jellyfish software (lines 262 – 264). We have also added information about karyotype, including counts of metacentric and acrocentric chromosomes (lines 260 -262). Satellite regions were not identified. That work will require future targeted analysis. Finally, we have now used FRC_align (FRCBam) to compare our new assembly to the older MesAur1.0 (lines 282 – 284 and Supplementary Figure 1).

Section titled "Transcript and protein alignments and annotation comparisons": How many INDELs were identified in the alignments of RNA-seq transcripts to the BCM_Maur_2.0 assembly? Was this count different from those discovered in the short read assembly?

We have now added (lines 319 – 322) the number of RefSeq gene models that required sequence gap fills in BCM_Maur_2.0 (33 fills) as compared to MesAur1.0 (5,050 fills). This is clearly a substantial improvement.

Section titled "Interferon type 1 alpha gene cluster": Were there any gaps that spanned the gene cluster or flanked it?

We have added a statement to the final paragraph of the Results section (lines 359 - 362) indicating that the interferon type I alpha gene cluster in our new assembly lies within more than 12 Mbp of contiguous genomic sequence with the nearest flanking gaps located 2.66 Mbp proximal and 9.07 Mbp distal to the Ifne and Ifnb1 genes, respectively.

Reviewer #2

Harris et al. present a high-quality Syrian hamster assembly BCM_Maur_2.0 with ONT long reads, Illumina short reads and Bionano optical map. They performed comprehensive comparison between the published hamster assembly MesAur1.0 and the new assembly, and further performed preliminary analysis to the interferon alpha I cluster. The discussion summarized the study well and provided possible further research fields. The work can undoubtedly contribute to the evolution and long disease studies. The whole paper is clearly described and easy to read. Thus, I think the paper is worthy for publication.

We thank the reviewer for this positive appraisal.

Below are some minor questions and suggestions for the paper:

1. Why were gaps introduced after Pilon polishing, according to the number in Table 1?

We are afraid there is some confusion here and we apologize if the initial text and table was not clear. There were no gaps introduced by the Pilon polishing step, which only

	corrects errors in basepair calls. The Bionano optical mapping step did identify a number of mis-joins in the initial scaffolding and therefore we broke some candidate scaffolds, creating separation among them. Please see Table 1 and lines 202 – 204).<br><br>2. When performing the assembly comparisons, the authors showed BCM_Maur_2.0 has much less gap than MesAur1.0. Could the authors show the number of genes that is rescued from the gap? This could further prove the advantage of the new assembly.<br><br>We agree with the reviewer that the number of gene models rescued or completed through the new assembly is an important issue. We feel that the BUSCO analysis is our best approach to assessing the degree of improvement. We found that the proportion of missing genes drops from 5.1% in MesAur1.0 to 3.2% in BCM_Maur_2.0 while the proportion of complete BUSCO genes increases from 89.0% in the earlier assembly to 93.9% in the new one. This information and further related statistics are provided in Table 2.<br><br>3. The author wrote "The NCBI annotations characterize twelve of these genes as interferon alpha- 12-like with predicted coding sequences of 190 amino acids (Supplemental Table 1).", but in the table gene LOC101824534 encodes protein with only 183aa. I suggest rewording the sentence, for example, remove the length description. Besides, in the paper the author preliminarily studied the interferon alpha genes. It would be better to be careful about the pseudogenes in further analysis, to avoid false positive caused by ONT sequencing errors which are not corrected by the Illumina short reads with Pilon.<br><br>We thank the reviewer for these suggestions and have made these changes in the text (lines 352 – 353).<br><br>4. Gene name in Fig. 3 should be italic. And it's better to also show the ifn1 alpha cluster of MesAur1.0 in the plot as well for a clear comparison between the assemblies.<br><br>We have italicized gene names and have added the MesAur1.0 chromosome region to Figure 3.<br><br>5. Version of some softwares such as Pilon and Assemblytics are missing<br><br>We now indicate in the Genome Assembly section that we used Pilon version 1.23 (line 165) and in the Quality Assessment section that we used Assemblytics version 1.2.1 (line 212). We have checked other software tools and have included version numbers if they were missing in the first submission.
**Additional Information:**	
**Question**	**Response**
Are you submitting this manuscript to a special series or article collection?	No
**Experimental design and statistics**<br><br>Full details of the experimental design and statistical methods used should be given in the Methods section, as detailed in our Minimum Standards Reporting Checklist. Information essential to interpreting the data presented should be made available in the figure legends.<br><br>Have you included all the information	Yes

requested in your manuscript?	
**Resources**<br><br>A description of all resources used, including antibodies, cell lines, animals and software tools, with enough information to allow them to be uniquely identified, should be included in the Methods section. Authors are strongly encouraged to cite Research Resource Identifiers (RRIDs) for antibodies, model organisms and tools, where possible.<br><br>Have you included the information requested as detailed in our Minimum Standards Reporting Checklist?	Yes
**Availability of data and materials**<br><br>All datasets and code on which the conclusions of the paper rely must be either included in your submission or deposited in publicly available repositories (where available and ethically appropriate), referencing such data using a unique identifier in the references and in the “Availability of Data and Materials” section of your manuscript.<br><br>Have you have met the above requirement as detailed in our Minimum Standards Reporting Checklist?	Yes

Manuscript Click here to access/download;Manuscript;Hamster_manu revision 102821_final.docx

Construction of a new chromosome-scale, long-read reference genome assembly for the Syrian hamster, *Mesocricetus auratus*

R. Alan Harris[1] rharris1@bcm.edu

Muthuswamy Raveendran[1] raveendr@bcm.edu

Dustin T. Lyfoung[2] lyfoung@wisc.edu

Fritz J Sedlazeck[1] fritz.sedlazeck@bcm.edu

Medhat Mahmoud[1] helmy.medhat@gmail.com

Trent M. Prall[3] prall@wisc.edu

Julie A. Karl[3] jakarl@wisc.edu

Harshavardhan Doddapaneni[1] doddapan@bcm.edu

Qingchang Meng[1] qingchang.meng@bcm.edu

Yi Han[1] yhan@bcm.edu

Donna Muzny[1] donnam@bcm.edu

Roger W. Wiseman[2, 3] rwwiseman@wisc.edu

David H. O'Connor[2, 3] dhoconno@wisc.edu

Jeffrey Rogers[1] jr13@bcm.edu

(Corresponding author: jr13@bcm.edu; 713-798-7783)

[1]Human Genome Sequencing Center and Department of Molecular and Human Genetics, Baylor College of Medicine, Houston, TX 77030

[2]Wisconsin National Primate Research Center, University of Wisconsin, Madison, WI 53711

[3]Department of Pathology and Laboratory Medicine, University of Wisconsin, Madison, WI 53711

Abstract

Background

The Syrian hamster (*Mesocricetus auratus*) has been suggested as a useful mammalian model for a variety of diseases and infections, including infection with respiratory viruses such as SARS-CoV-2. The MesAur1.0 genome assembly was generated in 2013 using whole-genome shotgun sequencing with short-read sequence data. Current more advanced sequencing technologies and assembly methods now permit the generation of near-complete genome assemblies with higher quality and greater continuity.

Findings

Here, we report an improved assembly of the *M. auratus* genome (BCM_Maur_2.0) using Oxford Nanopore Technologies long-read sequencing to produce a chromosome-scale assembly. The total length of the new assembly is 2.46 Gbp, similar to the 2.50 Gbp length of a previous assembly of this genome, MesAur1.0. BCM_Maur_2.0 exhibits significantly improved continuity with a scaffold N50 that is 6.7 times greater than MesAur1.0. Furthermore, 21,616 protein coding genes and 10,459 noncoding genes are annotated in BCM_Maur_2.0 compared to 20,495 protein coding genes and 4,168

noncoding genes in MesAur1.0. This new assembly also improves the unresolved regions as measured by nucleotide ambiguities, where approximately 17.11% of bases in MesAur1.0 were unresolved compared to BCM_Maur_2.0 in which the number of unresolved bases is reduced to 3.00%.

Conclusions

Access to a more complete reference genome with improved accuracy and continuity will facilitate more detailed, comprehensive, and meaningful research results for a wide variety of future studies using Syrian hamsters as models.

Keywords

Syrian hamster, *Mesocricetus auratus*, genome, disease model, COVID-19

Data Description

Introduction

The Syrian hamster (*Mesocricetus auratus*, NCBI:txid10036) has been used in biomedical research for decades because it is a good model for studies of cancer [1], reproductive biology [2] and infectious diseases [3,4], including SARS-CoV-2, influenza virus, and Ebola virus [5–9]. The use of Syrian hamsters in research has declined [10], likely due to advances in the genetic and molecular tools available for other rodents, especially laboratory mice, and not to a reduction in the utility of hamsters in biomedical research [3].

Syrian hamsters are particularly important for COVID-19 research. They spontaneously develop more severe lung disease than other animal models, such as wild-type mice, macaques, marmosets, and ferrets [5,11–14]. After intranasal infection, Syrian hamsters consistently show signs of respiratory distress, including labored breathing, but typically recover after 2 weeks [15]. This is in stark contrast to wild-type laboratory mice that are minimally susceptible to most SARS-CoV-2 strains that were circulating in 2020, though laboratory mice may be more susceptible to certain variants of concern that began circulating in 2021 [8,16]. Furthermore, a recent analysis has suggested that Syrian hamsters fed a high-fat, high-sugar diet exhibit accelerated weight gain and pathological changes in lipid metabolism, as well as more severe disease outcomes when subsequently infected with SARS-CoV-2 [17]. This result has obvious parallels with observations of the effects of comorbidities in humans suffering from COVID-19.

COVID-19 pathology in Syrian hamsters appears to be due to a dysregulated innate immune response involving signal transducer and activator of transcription factor 2 (STAT2)-dependent type I (IFN-I) and type III interferon (IFN-III) signaling [18]. IFN-I signaling can limit virus replication and dissemination and it has been shown that intranasal administration of IFN-I in Syrian hamsters reduces viral load and tissue damage [19]. The human angiotensin-converting enzyme 2 (ACE2) was identified as the cell entry receptor of SARS-CoV-2 [20]. In addition, upon the engagement of ACE2 with SARS-CoV2, cellular transmembrane protease 'serine 2' (TMPRSS2) mediates the priming of viral spike (S) protein by cleaving at the S1/S2 site and inducing the fusion of viral and host cellular membranes, thus facilitating viral entry into the cells [21]. Human ACE2 and hamster ACE2 receptors had previously been shown to share substantial sequence homology, which strongly points to interaction with SARS-CoV-2 receptor binding domain (RBD) structures and similar binding affinity [22]. *In silico* interaction prediction analysis suggests that human and hamster TMPRSS2 are structurally very similar. Even with slight differences in amino acid residue interactions, human and hamster TMPRSS2 activity are identical for residue interactions related to SARS-CoV-2 infectivity [22]. As COVID-19 causes systemic disease in people, precision modeling of specific aspects of pathogenesis will require carefully evaluating similarities and differences across various biological processes in humans and Syrian hamsters which, in turn, will require extensive genomic comparisons between the two species.

The currently available reference genome sequence for the Syrian hamster was produced in 2013 using a whole-genome shotgun sequencing approach implementing short read sequencing technology. The resulting MesAur1.0 reference sequence (Genbank

accession number GCA_000349665.1) is typical of those produced at that time, containing 237,699 separate contigs with contig N50 of 22,512 bp. The quality and research potential of the existing Syrian hamster genome is limited by the technology that was available at the time of its development; for example, the cluster of type I interferon genes was not resolvable with this technology. In this Data Note, we report the production of a new Syrian hamster reference genome that was sequenced using long-read methods on the Oxford Nanopore Technologies (ONT) PromethION platform and assembled into highly contiguous chromosomes using a combination of Flye [23] and Pilon [24] assembly software. The final assembly, BCM_Maur_2.0, improves upon quality and contiguity in comparison with MesAur1.0, with longer contigs and more contiguous sequence, allowing for a more complete reference genome with improved accuracy that will benefit a wide variety of future studies using the Syrian hamster reference genome.

Methods

DNA isolation, library construction, and sequencing

All genomic DNAs for this study were isolated from a single female LVG Golden Syrian hamster (SY011) that was purchased from Charles River, Inc. (Kingston, NY). All procedures were performed in accordance with the guidelines set by the Institutional Animal Care and Use Committee at the University of Wisconsin-Madison. The protocol was approved by the Institutional Animal Care and Use Committee at the University of Wisconsin-Madison (protocol number V00806). Data from this individual are available in NCBI BioProject PRJNA705675, BioSamples SAMN18096087 and SAMN18096088. Qiagen AllPrep DNA/RNA Mini kits were used to extract DNA from frozen liver while

Qiagen Blood and Cell Culture DNA Midi Kits were used for extractions from frozen kidney. Ultra-high molecular weight DNA for optical mapping was purified from frozen liver using an Animal Tissue DNA Isolation Kit from Bionano Genomics, Inc. (San Diego, CA).

Oxford Nanopore long-read sequencing

We prepared three separate genomic DNA isolates from the same Syrian hamster (BioSample SAMN18096087). These aliquots were sheared to distinct target fragment lengths (10 kb, 20kb and 30kb) in order to assess the effect of fragment size on flowcell yield and improve efficiency. The two smaller length fragment libraries were sheared using Covaris gTube and the 30kb targeted size library was fragmented with Diagnode Megarupter 3, all following manufacturer’s recommendations. The Oxford Nanopore sequencing libraries were prepared using the ONT 1D sequencing by ligation kit (SQK-LSK109). Briefly, 1-1.5ug of fragmented DNA was repaired with the NEB FFPE repair kit, followed by end repair and A-tailing with the NEB Ultra II end-prep kit. After a clean up step using AMPure beads, the prepared fragments were ligated to ONT specific adapters via the NEB blunt/TA master mix kit. The library underwent a final clean up and was loaded onto a PromethION flow cell per manufacturer's instructions. One library was sequenced per flow cell with standard parameters for 72 hrs. Base-calling was done onboard the PromethION instrument using neuronal network based software (Oxford Nanopore Technologies, UK).

Illumina sequencing

500ng of input genomic DNA from a kidney sample (BioSample SAMN18096088) was used to generate standard PCR-free Illumina paired-end sequencing libraries. Libraries

were prepared using KAPA Hyper PCR-free library reagents (KK8505, KAPA Biosystems) in Beckman robotic workstations (Biomek FX and FXp models). Total genomic DNA was sheared into fragments of approximately 200-600 bp in a Covaris E220 system (96-well format) followed by purification of the fragmented DNA using AMPure XP beads. A double size selection step was employed, with different ratios of AMPure XP beads, to select a narrow size band of sheared DNA molecules for library preparation. DNA end-repair and 3'-adenylation were then performed in the same reaction followed by ligation of the barcoded adaptors to create PCR-Free libraries. The resulting libraries were evaluated using the Fragment Analyzer (Advanced Analytical Technologies, Ames, Iowa) to assess library size and presence of remaining adaptor dimers. This was followed by qPCR assay using KAPA Library Quantification Kit and their SYBR FAST qPCR Master Mix to estimate the size and quantify fragment yield.

Sequencing was performed on the NovaSeq 6000 Sequencing System using the S4 reagent kit (300 cycles) to generate 2 x 150 bp paired-end reads. The final concentration of the libraries loaded on flowcells was 400-450 pM. Briefly, the libraries were diluted in an elution buffer and denatured in sodium hydroxide. The denatured libraries were loaded into each lane of the S4 flow cell using the NovaSeq Xp Flow Cell Dock. Each lane included ~1% of a PhiX control library for run quality control.

Genome Assembly

We generated 221 gigabases of sequence data using the ONT PromethION platform (NCBI BioProject PRJNA705675, SRA Experiment SRX11206953). This represents an anticipated 88X coverage of the expected 2.5 Gbp Syrian hamster genome. The raw

sequencing reads exhibited an N50 length of 15,730 bp. We used the Flye assembler v2.8.1 [23] to generate an initial *de novo* genome assembly. Given the potential sequence error rate of PromethION reads, it is advisable to use higher quality Illumina short reads mapped to an assembly to correct sequence errors in initial contigs. Consequently, we used Pilon software v. 1.23 [24] with default settings and 30X genome coverage of Illumina data (SRX10928323) generated from a kidney sample (SAMN18096088) obtained from the same individual for this sequence polishing step. Pilon sequence polishing was performed one time prior to the optical mapping analyses.

Optical mapping for scaffold improvement

Ultra-high molecular weight (UHMW) DNA was extracted following manufacturer's guidelines (Bionano Prep SP Tissue and Tumor DNA Isolation protocol) from frozen liver tissues obtained from the same animal used for ONT PromethION sequencing (SAMN18096087). Briefly, a total of 15-20mg of liver tissue was homogenized in cell buffer and digested with Proteinase K. DNA was precipitated with isopropanol and bound with nanobind magnetic disk (Bionano Genomics, USA). Bound UHMW DNA was resuspended in the elution buffer and quantified with Qubit dsDNA assay kits (ThermoFisher Scientific). DNA labeling was performed following manufacturer's protocols (Bionano Prep Direct Label and Stain protocol). Direct Labeling Enzyme 1 (DLE-1) reactions were carried out using 750 ng of purified UHMW DNA. Labeled DNA was loaded on Saphyr chips for imaging. The fluorescently labeled DNA molecules were imaged sequentially across nanochannel arrays (Saphyr chip) on a Saphyr instrument (Bionano Genomics Inc, USA). Effective genome coverage of greater than 100X was

achieved for all samples. All samples also met the following QC metrics: labelling density of ~15/100 kbp; filtered (>15kbp) N50 > 230 kbp; map rate > 70%.

Genome analysis of the resulting data was performed using software solutions provided by Bionano Genomics Inc. Briefly, automated optical genome mapping specific pipelines consisting of Bionano Access v1.4.3 and Bionano Solve v. 3.6.1 were used for data processing (BioNano Access Software User Guide). Hybrid scaffolding was performed using Bionano's custom software program implementing the following steps: 1) generate *in silico* maps for sequence assembly; 2) align *in silico* sequence maps against Bionano genome maps to identify and resolve potential conflicts in either data set; 3) merge the non-conflicting maps into hybrid scaffolds; 4) align sequence maps to the hybrid scaffolds; and 5) generate AGP and FASTA files for the scaffolds. Pairwise comparisons of all DNA molecules were made to generate the initial consensus genome maps (*.cmap). Genome maps were further refined and extended with best matching molecules. Optical map statistics were generated using Bionano software producing the Bionano Molecule Quality Report (MQR).

The optical map N50 (including only maps >=150 kbp and minSites >= 9) was 0.2341 Mbp and the average label density (scaffolds >= 150 kbp) was 17.40/100 kbp. This yielded an effective molecule coverage with optical mapping information of 125.38X. The optical mapping analysis identified 84 conflicts with the prior Flye/Pilon scaffolds and these initial scaffolds were broken at those 84 sites. The completed assembly was submitted to NCBI and is available under accession GCA_017639785.1.

Gene annotation

NCBI performed gene annotation using RNA-Seq data including data consisting of multiple tissues including lung, trachea, brain, olfactory bulb and small intestine that are targets for SARS-CoV-2 infection (NCBI BioProject PRJNA675865) [19].

Quality assessment

To assess the quality of our assembly compared to the previous MesAur1.0 we used Quast v5.0.2 [25] together with MUMmer v3.23 [26] and Assemblytics v. 1.2.1 [27]. These tools provided a detailed comparison between these assemblies. In addition, the Illumina reads from the original reference (NCBI SRA SRR413408) were mapped to our assembly and the MesAur1.0 reference using BWA v0.7.17 [28]. Quast was used to obtain discordant pair statistics.

We next used the software Benchmarking Universal Single-Copy Orthologs (BUSCO) v3.0.2 [29] to assess the quality of the genome assembly. BUSCO is based on the concept that single-copy orthologs should be highly conserved among closely related species. BUSCO performs gene annotation on an assembly and reports the number of gene models generated. BUSCO was performed using the OrthoDB v9 (odb9) release consisting of 6192 genes shared across the superorder Euarchontoglires [30], the appropriate test for the Syrian hamster.

In addition, FRC^{bam} [31] was used to compute Feature Response Curves (FRCurve) from the alignment of Illumina reads to the assembled contigs. FRC v1.3.0 was employed to evaluate both assemblies, using default parameters. BCM_Maur_2 was further evaluated

using paired end mappings of the Illumina reads that had been used for Pilon polishing (SRX10928323). MesAur1.0 was then similarly evaluated using paired end mappings of Illumina reads used for the MesAur1.0 assembly (SRR413408).

Results

The initial Flye assembly consisted of 2.38 Gbp of sequence across 6,741 scaffolds with a scaffold N50 of 10.56 Mbp (**Table 1**). Pilon polishing of the Flye assembly had little effect on these metrics but significant improvements were obtained when Bionano optical mapping results were used to improve scaffolding. As shown in **Table 1**, the optical mapping step reduced the total number of scaffolds in the final assembly by 395 (5.9%) while increasing the N50 scaffold length by more than 8-fold to 85.18 Mbp.

Of the 6192 BUSCO gene models, 88.91% were annotated as complete genes in the initial Flye assembly (**Table 2**). Pilon polishing of this Flye-alone assembly added another 295 genes annotated completely and increased this proportion to 93.67% of the BUSCO gene model dataset. Improvements in assembly scaffolding resulting from the Bionano optical mapping step together with Pilon error correction decreased the proportions of fragmented and missing BUSCO gene models in the new assembly to 2.9% and 3.2% respectively, also improvements over the MesAur1.0 assembly. This advance translates to an additional 305 complete BUSCO genes identified in the new assembly.

Table 1. Assembly statistics for BCM_Maur_2.0 versus the MesAur1.0 Syrian hamster assembly

Parameter	MesAur1.0	Flye	Flye + Pilon	Flye + Pilon + Bionano (BCM_Maur_2.0)
Assembly length (bp)	2,504,908,775	2,381,258,546	2,383,228,608	2,457,062,007
Ungapped length (bp)	2,076,159,990	2,381,254,546	2,383,226,373	2,383,228,883
Number of scaffolds	21,483	6,741	6,741	6,346
N50 scaffold length (bp)	12,753,307	10,564,357	10,573,641	85,184,847
Number of contigs	237,699	6,781	6,779	7,057
N50 contig length (bp)	22,512	10,022,145	10,097,207	9,471,653

Table 2. BUSCO statistics for BCM_Maur_2.0 versus the MesAur1.0 Syrian hamster assembly

	MesAur1.0	Flye	Flye + Pilon	Flye + Pilon + Bionano (BCM_Maur_2.0)
Complete[a]	88.97%	88.91%	93.67%	93.90%
Complete and single-copy	88.47%	88.05%	92.80%	93.12%
Complete and duplicated	0.50%	0.86%	0.87%	0.78%
Fragmented	5.89%	5.99%	3.04%	2.87%
Missing	5.14%	5.10%	3.29%	3.23%

[a]6192 gene models were included in this analysis

Assembly Comparisons

We also performed additional comparisons between the two assemblies. As background, the karyotype of *M. auratus* is diploid 2n = 44, including 14 pairs of metacentric chromosomes, 3 pairs of telocentrics and 5 pairs of acrocentrics [32]. Illumina read k-mer analyses were performed to estimate the genome size using SGA preqc [33] (2.57 Gbp) and Jellyfish [34] (2.90 Gbp). The total length of the BCM_Maur_2.0 assembly is 2.46 Gbp compared to the previous version's 2.50 Gbp. Despite having a similar total length, BCM_Maur_2.0 shows an improved continuity with a scaffold N50 that is 6.7 times greater than MesAur1.0 (**Table 1**); the L50 (i.e. the number of contigs longer than or equal to the N50 length) of BCM_Maur_2.0 is 22 compared to MesAur1.0's 121. The longest scaffold

of BCM_Maur_2.0 (187 Mb) is 2.35 times larger than the longest scaffold from the previous assembly. N50 is calculated in the context of the assembly size rather than the genome size, so the NG50 statistic was used to directly compare the different assemblies. NG50 is the same as N50 except that it reports the length of the contig at which the size-ordered contigs (longest to shortest) collectively reaches 50% of the known or estimated genome size [35]. **Figure 1** illustrates the improved cumulative contig sequence length for any given NG50 value that is generated from the BCM_Maur_2.0 assembly as compared to MesAur1.0. The BCM_Maur_2.0 assembly further improves the unresolved regions as measured by nucleotide ambiguities (i.e. number of N's included in the final contigs). Approximately 17.11% of bases in MesAur1.0 were unresolved. BCM_Maur_2.0 reduces the number of unresolved bases to 3.00%, with only very small gaps throughout the entire genome. **Figure 2** displays the overall increase in continuity of the BCM_Maur_2.0 assembly with longer contigs than the MesAur1.0 assembly and fewer short contigs. Finally, we compared feature response curves for BCM_Maur_2.0 and MesAur1.0 using FRC^{Bam} [31]. FRC^{Bam} shows that our new assembly is substantially more accurate based on the feature response approach (Supplementary Figure 1).

To establish the correctness of the structure and completeness of BCM_Maur_2.0, we also leveraged the Illumina short-reads that were published as part of the MesAur1.0 assembly project. When mapping the MesAur1.0 Illumina reads back to the MesAur1.0 reference, only 92.19% reads mapped successfully. When the same Illumina reads were instead mapped to BCM_Maur_2.0, 97.32% mapped successfully. When considering only properly paired reads, 75.76% and 87.62% mapped to MesAur1.0 and BCM_Maur_2.0, respectively.

Alignments between the current and previous Syrian hamster assemblies performed by NCBI [36] show that BCM_Maur_2.0 covers 98.95% of MesAur1.0 while MesAur1.0 only covers 86.67% of BCM_Maur_2.0. This together with the additional 307 Mbp of ungapped sequence in BCM_Maur_2.0 indicates that BCM_Maur_2.0 is a more complete representation of the Syrian hamster genome. The percent identity in the regions aligned between the two assemblies is 99.76%.

Transcript and Protein Alignments and Annotation Comparisons

NCBI annotation of BCM_Maur_2.0 [36] with Syrian hamster transcript and protein data show this assembly to be of high quality. Transcript alignments of Syrian hamster RefSeq (n=273), Genbank (n=751), and EST (n=558) data to BCM_Maur_2.0 show 99.44% or more average percent identity and 98.88% or more average percent coverage. Alignments of these same transcript datasets to MesAur1.0 show 99.13% or more average percent identity and 93.49% or more average percent coverage. Protein alignments of Syrian hamster RefSeq (n=261) and Genbank (n=485) data to BCM_Maur_2.0 show 80.95% or more average percent identity and 89.18% or more average percent coverage. Alignments of these same protein datasets to MesAur1.0 show 80.57% or more average percent identity and 84.87% or more average percent coverage.

NCBI annotated 21,616 protein coding genes and 10,459 noncoding genes in BCM_Maur_2.0 compared to 20,495 protein coding genes and 4,168 noncoding genes

in MesAur1.0 [37]. Only 7% of gene annotations are identical between BCM_Maur_2.0 and MesAur1.0, suggesting that a number of previous errors have been corrected, though some differences are likely to be real differences between the animals used for the different assemblies. Minor changes between BCM_Maur_2.0 and MesAur1.0 were made in 46% of gene annotations and major changes were made in 15% of gene annotations. We further note that, based on NCBI annotation feature counts, BCM_Maur_2.0 has only 33 RefSeq models that were filled using transcript sequence to compensate for an assembly gap [36]. This is compared to 5,050 RefSeq models similarly compensated in MesAur1.0.

Interferon type 1 alpha gene cluster

Given the importance of type I interferon responses during SARS-CoV-2 infection, we next compared the interferon type I alpha gene cluster in the BCM_Maur_2.0 assembly relative to this genomic region in the original MesAur1.0 assembly. The MesAur1.0 scaffold NW_004801649.1 includes annotations for four interferon type I alpha loci but this genomic sequence is riddled with numerous gaps. Of these four candidate loci, only LOC101824534 appears to contain a complete interferon alpha-12-like coding sequence with the ability to encode a predicted protein (XP_005074343.1). The LOC101824794 gene sequence can only encode a 162 amino acid protein due to a 5’ truncation. The remaining pair of candidate genes (LOC101836618 and LOC101836898) appear to have aberrant transcript models that have fused putative exons from neighboring loci. In mice and humans, the interferon alpha gene cluster is flanked by single copy interferon beta 1 (*Ifnb1*) and interferon epsilon (*Ifne*) genes. Although neither of these genes are present on the MesAur1.0 scaffold NW_004801649.1, this assembly does contain a *Ifne* gene on

a short 2,408 bp contig that is predicted to code for a protein of 192 amino acids. These observations emphasize the need for an improved genomic assembly for Syrian hamsters given that the interferon alpha gene cluster includes more than a dozen tightly linked functional genes plus multiple pseudogenes in a wide variety of species including mice and humans.

In the BCM_Maur_2.0 assembly, the interferon type I alpha gene cluster is contained on the NW_024429197.1 super scaffold that spans nearly 75 Mbp. **Figure 3** illustrates this genomic region in comparison with the interferon type 1 alpha regions of MesAur1.0 (NW_004801649.1) and the well-characterized C57BL/6J mouse assembly (NC_000070.7). Fourteen predicted interferon type I alpha genes as well as five presumptive pseudogenes lie within a span of 196 Kbp of the new Syrian hamster assembly (**Figure 3** and **Supplemental Table 1**). This genomic organization is quite comparable to that observed in the mouse genome where there are also fourteen functional interferon alpha genes and four pseudogenes. This hamster gene cluster is flanked by *Ifnb1* and *Ifne* genes consistent with expectations from the mouse and other species. The NCBI annotations characterize twelve of these genes as interferon alpha-12-like (**Supplemental Table 1**). The remaining pair of functional genes (LOC101824794 and LOC121144100) are listed as interferon alpha-9-like and they encode shorter predicted proteins. The increased length of this genomic region in the mouse assembly is largely due to the presence of the interferon zeta gene family (*Ifnz*, Gm13271, Gm13272, etc.). This *Ifnz* gene family appears to be absent in Syrian hamsters since the closest matches to predicted hamster protein sequences are only 28% identical at the amino acid level. The interferon type I alpha gene cluster in the BCM_Maur_2.0 assembly

lies within more than 12 Mbp of contiguous genomic sequence with the nearest flanking gaps located 2.66 Mbp proximal and 9.07 Mbp distal to the *Ifne* and *Ifnb1* genes, respectively. The availability of a contiguous hamster genomic sequence and associated transcriptional regulatory elements for this complex immune gene region may be helpful for investigators who are interested in unravelling mechanisms that control interferon expression during infections with SARS-CoV-2 as well as challenges with other viral pathogens.

Conclusions

The improved Syrian hamster assembly and annotation described here will facilitate research into this important animal model for COVID-19. Specifically, reagents for studying immune re sponses in hamsters have lagged behind those available for laboratory mice. BCM_Maur_2.0 will facilitate the identification of cross-reactive reagents originally developed to study immunity in other species. Additionally, a more accurate genome assembly will improve the analyses of host responses to infection by enabling more accurate interpretation of RNA-seq experiments.

Relative to other recent assemblies that use a combination of long-read sequencing and short-read polishing, this genome assembly and annotation compares very favorably. The scaffold N50 of >85 Mbp is quite consistent with other long read assemblies. The contig N50 and total number of scaffolds or contigs are likewise reasonable and consistent with other similar mammalian reference genomes. The number of protein coding genes identified is within the expected range, although additional attention will likely be needed

to resolve duplicated, repetitive gene loci, potentially leveraging recent advances in ultralong read sequencing.

What additional genomic resources would be needed to make hamsters a better model for COVID-19? Deep long read transcriptome analysis of multiple tissues and ages would be the best next step, in order to define not just the genes expressed but the alternative splicing of genes across tissues and developmental stages. Also, long read RNA-seq of tissues following experimental challenge with SARS-CoV-2 and other viruses would facilitate improvements in the quality of antiviral gene models.

The availability of higher accuracy sequences should lead to the development of specific reagents for monitoring immune responses. For example, epitopes that are shared between hamsters and other rodents can be used to identify monoclonal antibody reagents for flow cytometry that are predicted to be cross-reactive. Additional reagent development will be enabled by creating synthetic versions of hamster proteins that can be used as immunogens to make hamster-specific antibodies.

One surprising motivation for this study is that Syrian hamsters, which were quickly identified as a high value model for COVID-19, did not have a higher quality reference genome at the start of the pandemic. While we worked quickly to generate this data and make it available to the scientific community, better preparedness will be critical for future unexpected epidemics. To this end, we would encourage investment in continued refinement and improvement of reference genomes for all of the rodent, bat and nonhuman primate models that are commonly used to study viruses in order to prevent

this situation from recurring in the future. Such an investment would also yield improved genomic resources that would provide broad benefit to the entire scientific community.

Availability of Supporting Data and Materials

The MesAur1.0 genome assembly is available in the NCBI database under BioProject PRJNA77669 (GenBank accession GCA_000349665.1). The new BCM_Maur_2.0 genome assembly is available in the NCBI data repository under BioProject PRJNA705675 (GenBank accession GCA_017639785.1). Oxford Nanopore (SRX11206953) and Illumina (SRX10928323) sequencing data are available through the NCBI SRA. The Illumina RNA-Seq data from multiple tissues including lung, trachea, brain, olfactory bulb and small intestine are available under NCBI BioProject PRJNA675865.

Additional Files

Supplementary Table 1. Predicted genes in the Interferon type 1 alpha cluster of the BCM_Maur_2.0 assembly.

Abbreviations

ACE2: angiotensin-converting enzyme 2; BCM: Baylor College of Medicine; bp: base pairs; BUSCO: Benchmarking Universal Single-Copy Orthologs; BWA: Burrows-Wheeler Aligner; COVID-19: coronavirus disease 2019; EST: expressed sequence tag; FFPE: formalin-fixed, paraffin-embedded; Gbp: gigabase pairs; GC: guanine-cytosine; IFN: interferon; kbp: kilobase pairs; Mbp: megabase pairs; MQR: Molecule Quality Report; NCBI: National Center for Biotechnology Information; NEB: New England BioLabs; ng: nanogram; ONT: Oxford Nanopore Technologies; PCR: polymerase chain reaction; RBD: receptor-binding domain; RNA-Seq: RNA-sequencing; SARS-CoV-2: severe acute respiratory syndrome coronavirus 2; STAT2: signal transducer and activator of transcription factor 2; TMPRSS2: transmembrane protease serine 2

Competing interests

The authors declare that they have no competing interests.

Funding

This research was supported by contract HHSN272201600007C awarded to DHO from the National Institute of Allergy and Infectious Diseases of the National Institutes of Health. The content of this publication is solely the responsibility of the authors and does not necessarily represent the official views of the National Institutes of Health.

Authors' Contributions

R.A.H. performed genome assembly and quality assessment, data and metadata submission, and contributed to manuscript preparation. F.S. and M.M. performed assembly assessment and comparison analyses. T.M.P. and R.W.W. performed transcript and annotation comparisons. D.H.O. managed experimental design and oversight and coordinated manuscript preparation. H.D., Q.M. and Y.H. developed, optimized and implemented protocols for ONT PromethION sequencing. M.R., D.M., J.A.K. and J.R. performed project and/or data management. R.A.H., D.H.O., D.T.L., T.M.P., R.W.W., M.M., F.S. and J.R. wrote the manuscript. All authors approved the manuscript.

Acknowledgements

We are extremely grateful to Dr. Tadashi Maemura for collecting the Syrian hamster tissues that were used for the sequence analyses described here. We also thank Dr. Benjamin tenOever for sharing Syrian hamster RNA-Seq datasets generated by his group prior to publication. And we also wish to thank two reviewers for their helpful comments.

Figure 1: Cumulative length and continuity comparison of MesAur1.0 and BCM_Maur_2.0. This summarizes the length of contigs/scaffolds across the assemblies. Given the length of contigs, the NG50 (mid x-axis) summarizes the sequence length of the shortest contig/scaffold at 50% of the total genome length. For genome length, the established MesAur1.0 was used.

Figure 2: Contig length and count comparison between BCM_Maur_2.0 and MesAur1.0. Log length of contigs on the X axis and normalized count on the Y axis comparing BCM_Maur_2.0 assembly and the previous assembly. Contigs from BCM_Maur_2.0 are shown red and contigs for MesAur1.0 are shown in gray.

Figure 3: Comparison of interferon type 1 alpha gene cluster between MesAur1.0, BCM_Maur_2.0 and GCRm39 mouse genome assembly. The genomic intervals illustrated here are defined by the flanking interferon beta 1 and interferon epsilon genes except for MesAur1.0 which does not include an interferon epsilon or beta 1 gene in a continuous sequence with interferon type 1 alpha genes. White space within each scaffold represents gaps in the MesAur1.0 assembly. Accession numbers for each genomic sequence are indicated on the right with genomic coordinates for the extracted intervals shown below their respective accession numbers. Predicted interferon type 1 alpha genes are highlighted in blue while putative pseudogenes are depicted with open symbols and labelled below each assembly.

References

1. LaRocca CJ, Han J, Gavrikova T, Armstrong L, Oliveira AR, Shanley R, et al.. Oncolytic adenovirus expressing interferon alpha in a syngeneic Syrian hamster model for the treatment of pancreatic cancer. *Surgery*. 157:888–982015;

2. Pal S, Haldar C, Verma R. Photoperiodic modulation of ovarian metabolic, survival, proliferation and gap junction markers in adult golden hamster, Mesocricetus auratus. *Comp Biochem Physiol A Mol Integr Physiol*. 263:1110832021;

3. McCann KE, Sinkiewicz DM, Norvelle A, Huhman KL. De novo assembly, annotation, and characterization of the whole brain transcriptome of male and female Syrian hamsters. *Sci Rep*. 7:404722017;

4. Saini S, Rai AK. Hamster, a close model for visceral leishmaniasis: Opportunities and challenges. *Parasite Immunol*. 42:e127682020;

5. Chan JF-W, Zhang AJ, Yuan S, Poon VK-M, Chan CC-S, Lee AC-Y, et al.. Simulation of the Clinical and Pathological Manifestations of Coronavirus Disease 2019 (COVID-19) in a Golden Syrian Hamster Model: Implications for Disease Pathogenesis and Transmissibility. *Clin Infect Dis*. 71:2428–462020;

6. Prescott J, Falzarano D, Feldmann H. Natural Immunity to Ebola Virus in the Syrian Hamster Requires Antibody Responses. *J Infect Dis*. 212 Suppl 2:S271–62015;

7. Huo J, Mikolajek H, Le Bas A, Clark JJ, Sharma P, Kipar A, et al.. A potent SARS-CoV-2 neutralising nanobody shows therapeutic efficacy in the Syrian golden hamster model of COVID-19. *Nat Commun*. 12:54692021;

8. Mohandas S, Yadav PD, Shete A, Nyayanit D, Sapkal G, Lole K, et al.. SARS-CoV-2 Delta Variant Pathogenesis and Host Response in Syrian Hamsters. *Viruses*. 2021; doi: 10.3390/v13091773.

9. Mifsud EJ, Tai CM, Hurt AC. Animal models used to assess influenza antivirals. *Expert Opin Drug Discov*. 13:1131–92018;

10. Gao M, Zhang B, Liu J, Guo X, Li H, Wang T, et al.. Generation of transgenic golden Syrian hamsters. *Cell Res*. 24:380–22014;

11. Imai M, Iwatsuki-Horimoto K, Hatta M, Loeber S, Halfmann PJ, Nakajima N, et al.. Syrian hamsters as a small animal model for SARS-CoV-2 infection and countermeasure development. *Proc Natl Acad Sci U S A*. 117:16587–952020;

12. Rockx B, Kuiken T, Herfst S, Bestebroer T, Lamers MM, Oude Munnink BB, et al.. Comparative pathogenesis of COVID-19, MERS, and SARS in a nonhuman primate model. *Science*. 368:1012–52020;

13. Rogers TF, Zhao F, Huang D, Beutler N, Burns A, He W-T, et al.. Isolation of potent SARS-CoV-2 neutralizing antibodies and protection from disease in a small animal model. *Science*. 369:956–632020;

14. Shi J, Wen Z, Zhong G, Yang H, Wang C, Huang B, et al.. Susceptibility of ferrets, cats, dogs, and other domesticated animals to SARS-coronavirus 2. *Science*. 368:1016–202020;

15. Muñoz-Fontela C, Dowling WE, Funnell SGP, Gsell P-S, Riveros-Balta AX, Albrecht RA, et al.. Animal models for COVID-19. *Nature*. 586:509–152020;

16. Montagutelli X, Prot M, Levillayer L, Salazar EB, Jouvion G, Conquet L, et al.. The B1.351 and P.1 variants extend SARS-CoV-2 host range to mice. bioRxiv.

17. Port JR, Adney DR, Schwarz B, Schulz JE, Sturdevant DE, Smith BJ, et al.. Western diet increases COVID-19 disease severity in the Syrian hamster. *bioRxiv*. 2021; doi: 10.1101/2021.06.17.448814.

18. Boudewijns R, Thibaut HJ, Kaptein SJF, Li R, Vergote V, Seldeslachts L, et al.. STAT2 signaling restricts viral dissemination but drives severe pneumonia in SARS-CoV-2 infected hamsters. *Nat Commun*. 11:58382020;

19. Hoagland DA, Møller R, Uhl SA, Oishi K, Frere J, Golynker I, et al.. Leveraging the antiviral type I interferon system as a first line of defense against SARS-CoV-2 pathogenicity. *Immunity*. 54:557–70.e52021;

20. Brooke GN, Prischi F. Structural and functional modelling of SARS-CoV-2 entry in animal models. *Sci Rep*. 10:159172020;

21. Hoffmann M, Kleine-Weber H, Schroeder S, Krüger N, Herrler T, Erichsen S, et al.. SARS-CoV-2 Cell Entry Depends on ACE2 and TMPRSS2 and Is Blocked by a Clinically Proven Protease Inhibitor. *Cell*. 181:271–80.e82020;

22. Rizvi ZA, Dalal R, Sadhu S, Kumar Y, Shrivastava T, Gupta SK, et al.. Immunological and cardio-vascular pathologies associated with SARS-CoV-2 infection in golden syrian hamster. Cold Spring Harbor Laboratory.

23. Kolmogorov M, Yuan J, Lin Y, Pevzner PA. Assembly of long, error-prone reads using repeat graphs. *Nat Biotechnol*. 37:540–62019;

24. Walker BJ, Abeel T, Shea T, Priest M, Abouelliel A, Sakthikumar S, et al.. Pilon: an integrated tool for comprehensive microbial variant detection and genome assembly improvement. *PLoS One*. 9:e1129632014;

25. Gurevich A, Saveliev V, Vyahhi N, Tesler G. QUAST: quality assessment tool for genome assemblies. *Bioinformatics*. 29:1072–52013;

26. Kurtz S, Phillippy A, Delcher AL, Smoot M, Shumway M, Antonescu C, et al.. Versatile and open software for comparing large genomes. *Genome Biol*. 5:R122004;

27. Nattestad M, Schatz MC. Assemblytics: a web analytics tool for the detection of variants from an assembly. *Bioinformatics*. 32:3021–32016;

28. Li H. Aligning sequence reads, clone sequences and assembly contigs with BWA-MEM. arXiv [q-bio.GN].

29. Seppey M, Manni M, Zdobnov EM. BUSCO: Assessing Genome Assembly and Annotation Completeness. *Methods Mol Biol*. 1962:227–452019;

30. : BUSCO. https://busco-archive.ezlab.org/v3/datasets/euarchontoglires_odb9.tar.gz Accessed 2021 Jun 4.

31. Vezzi F, Narzisi G, Mishra B. Reevaluating assembly evaluations with feature response curves: GAGE and assemblathons. *PLoS One*. 7:e522102012;

32. Lehman JM, Macpherson I, Moorhead PS. KARYOTYPE OF THE SYRIAN HAMSTER. *J Natl Cancer Inst*. 31:639–501963;

33. Simpson JT. Exploring genome characteristics and sequence quality without a reference. *Bioinformatics*. 30:1228–352014;

34. Marçais G, Kingsford C. A fast, lock-free approach for efficient parallel counting of occurrences of k-mers. *Bioinformatics*. 27:764–702011;

35. Alhakami H, Mirebrahim H, Lonardi S. A comparative evaluation of genome assembly reconciliation tools. *Genome Biol*. 18:932017;

36. : Mesocricetus auratus Annotation Report. https://www.ncbi.nlm.nih.gov/genome/annotation_euk/Mesocricetus_auratus/103/ Accessed 2021 Jun 4.

37. : Mesocricetus auratus Annotation Report. https://www.ncbi.nlm.nih.gov/genome/annotation_euk/Mesocricetus_auratus/102/ Accessed 2021 Jun 4.

Figure 1

Click here to access/download;Figure;Figure1.pdf

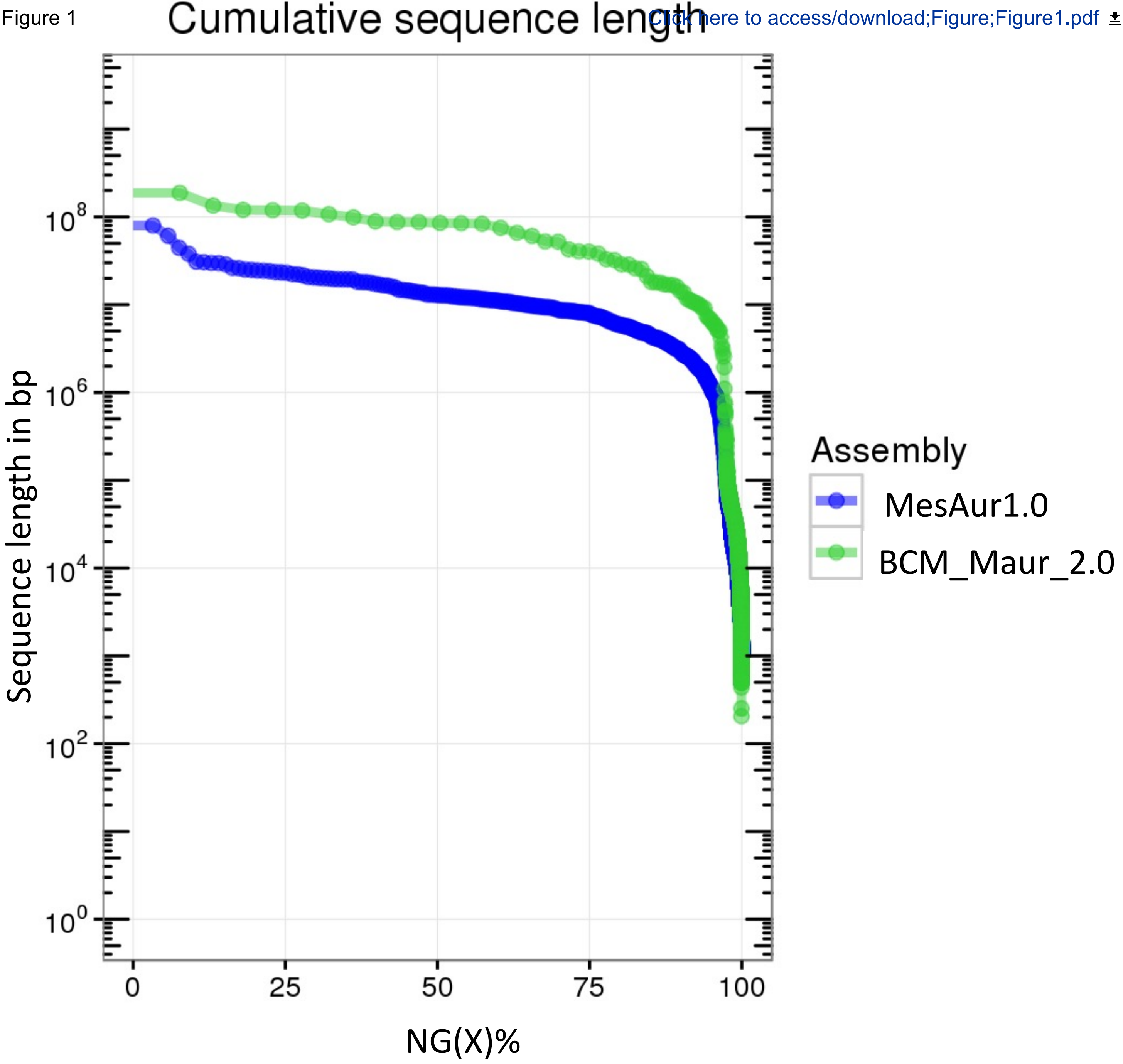

Figure 2

Click here to access/download;Figure;Figure2.pdf

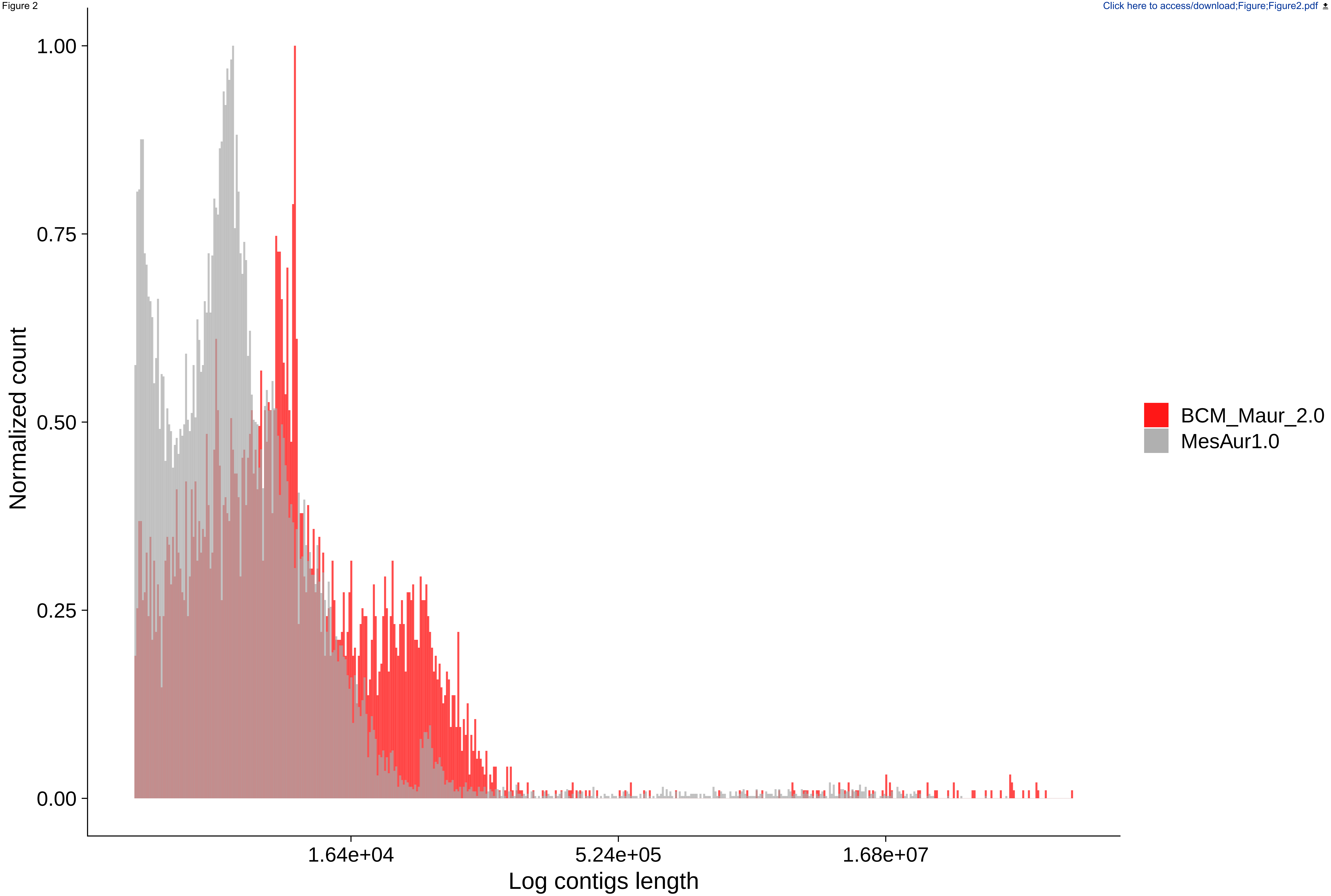

Figure 3

Click here to access/download;Figure;Figure3-10.7.21-revised.pdf

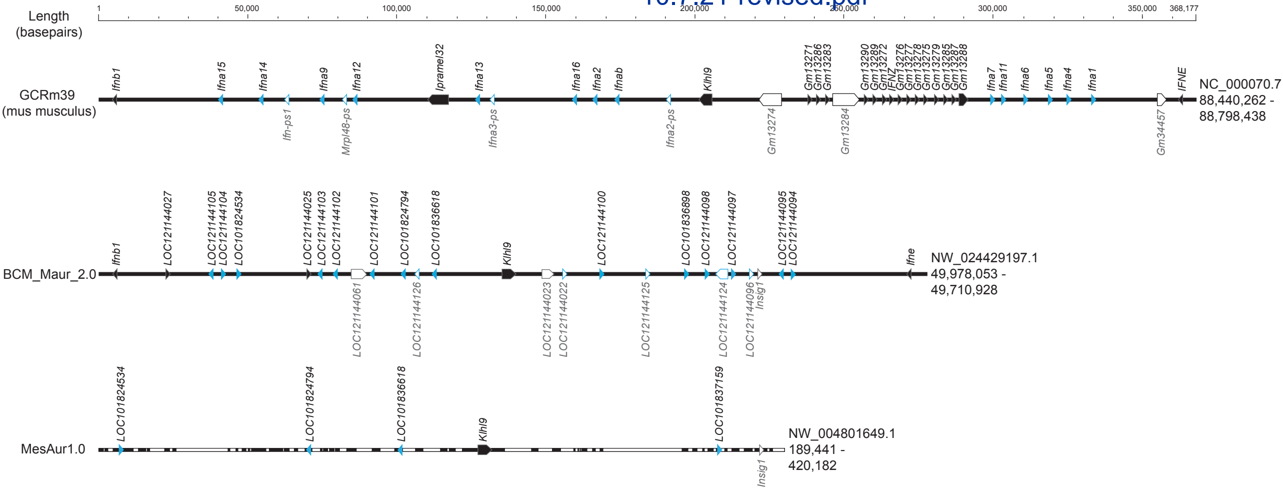

Click here to access/download
Supplementary Material
Supplemental Table 1. Interferon alpha genes.xlsx

Supplementary Figure

Click here to access/download
Supplementary Material
FRC_align.pdf

Personal Cover

Click here to access/download;Personal Cover;Gigascience revision cvr ltr 102921.pdf

Baylor College of Medicine
HUMAN GENOME SEQUENCING CENTER

ONE BAYLOR PLAZA
ALKEK BUILDING, 15th FLOOR
MS: BCM226
HOUSTON, TEXAS 77030

713-798-6539
713-798-5741 FAX

October 29, 2021

Scott Edmunds, Ph.D.
Editor-in-Chief
GigaScience

Dear Dr. Edmunds,

My colleagues and I are pleased to submit our revised manuscript titled "Construction of a new chromosome-scale, long read reference genome assembly for the Syrian hamster, *Mesocricetus auratus*," manuscript GIGA-D-21-00197. We have now completed our revisions, adding substantial new information as requested by the reviewers. We have also improved the clarity of the text through the editing of specific paragraphs, and we have added a new Supplementary Figure. We believe that we have addressed all the comments and concerns raised by the reviewers. In addition to the manuscript, we also submit a formal Response to Reviewers that details the specific changes made in relation to each reviewer comment.

We submit this revised manuscript in the hope that *GigaScience* will now find this acceptable for publication. We appreciate your efforts in this matter and look forward to your response.

With best wishes,

Jeffrey A Rogers

Jeffrey Rogers, Ph.D.
