# Supplementary figures and images for: Construction of a new chromosome-scale, long-read reference genome assembly for the Syrian hamster, Mesocricetus auratus
Source: Gigascience. 2022 May 28;11:giac039. doi: 10.1093/gigascience/giac039 (PMC9155146; doi:10.1093/gigascience/giac039)

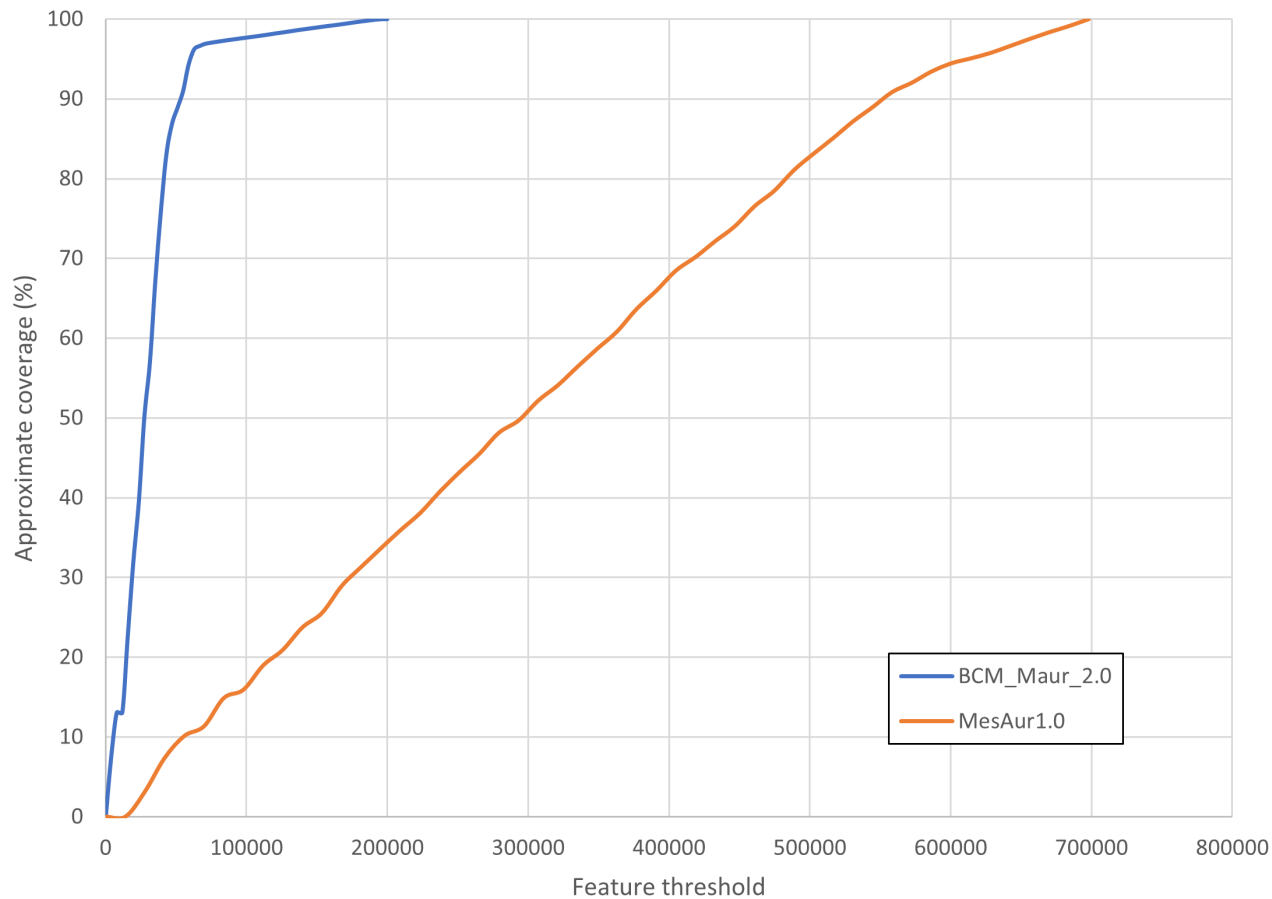

Supplement: giac039_Supplemental_Files [file giac039_supplemental_files.zip › FRC_align.pdf]
