# Supplementary material for: Construction of a new chromosome-scale, long-read reference genome assembly for the Syrian hamster, Mesocricetus auratus
Source: Gigascience. 2022 May 28;11:giac039. doi: 10.1093/gigascience/giac039 (PMC9155146; doi:10.1093/gigascience/giac039)
Supplement: giac039_Response_to_Reviewer_Comments_Original_Submission [file giac039_response_to_reviewer_comments_original_submission.pdf]

Author's Response To Reviewer Comments

Close

GIGA-D-21-00197
Harris et al.
Title: Construction of a new chromosome-scale, long read reference genome assembly for the Syrian hamster, Mesocricetus auratus

Response to Reviewer Critiques

We thank both reviewers for their largely positive evaluations of our manuscript. We have now addressed all the specific issues identified in their critiques, as outlined below. The reviewer comments are presented in Arial font and our responses are in Times New Roman font.

Reviewer #1

Summary: In this manuscript, Harris et al. detail the methods they used to create a new reference genome for the Syrian hamster, which is an important model for respiratory disease pathogens. They used several different sequencing technologies to generate the contigs and scaffolds for their new assembly, and achieved a relatively continuous end product. The analysis is suitable for the "genome report" style format (with one omission detailed below in my comments); however, the manuscript suffers from some awkward phrasing and grammar errors in the results and methods. I list my comments below in the relative order in which I encountered them in the manuscript. Since the authors did not provide line numbers in their submission, I provide my comments as a block listing of questions/suggestions/critiques.

We thank Reviewer #1 for their overall positive assessment of our manuscript.

Section titled "oxford nanopore long-read sequencing": The description of the shearing is awkward. I recommend revising the first sentence to state that the genomic DNA isolates were sheared to three lengths (without providing these lengths in the sentence). In subsequent sentences, provide the lengths in situ with the methods used to prepare them. Also, it is unclear why three different fragment lengths were used here for oxford nanopore sequencing. Given that these fragment lengths are relatively similar in size (e.g. not disparate lengths similar to recent ultra-long nanopore read preps of >100kb), it would be very helpful to the reader if justification was given for this approach.

We thank the reviewer for pointing out these issues with the text. We have now re-written this paragraph (lines 121 - 126) to increase clarity and have added the recommended explanations.

Section titled "Genome assembly": This entire paragraph is awkwardly phrased with numerous past- or present-tense changes. Additionally, the reference to the Pilon polisher needs to be cited, and details need to be provided on what settings were used for Pilon polishing (it is often recommended to correct only indels and to omit gap-filling) and how many iterations of polishing were used. Details are missing on how BioNano optical maps were generated, and what DNA was used as input in the process. Also, what software was used to compare BioNano optical maps, and with what settings? Finally, it appears that the RNA-seq data used by NCBI for annotation was used in another study. Citation to that study would be required so that the reader is aware that the data resulted from different individuals other than the reference individual sequenced in this analysis.

The section titled “Genome assembly” has now been extensively re-written (lines 158 – 168). We have added the citation to Pilon software and included more details on the analyses using Pilon (lines 164 – 168). We have also incorporated significantly more information about the methods used in the optical mapping analyses (lines 170 – 205) and have included the citation to the publication that reported the RNA-seq studies that provided the transcriptome data for annotation of this genome (line 209).

Section titled "Assembly Comparisons": What is the expected c-value of the Syrian Hamster genome? Also, what is the karyotype count? Are any of the chromosomes metacentric or acrocentric? Were any satellite regions identified and annotated in this assembly? Finally, I would have preferred that assembly comparisons be conducted with feature response curves, such as those produced by the program "FRC_align" as this provides a useful metric to assess assembly "correctness" by length.

Despite substantial effort, we cannot find information about the c-value for M. auratus. However, in lieu of c-value data, we now provide the results of k-mer analysis of genome length using both SGA preqc and Jellyfish software (lines 262 – 264). We have also added information about karyotype, including counts of metacentric and acrocentric chromosomes (lines 260 -262). Satellite regions were not identified. That work will require future targeted analysis. Finally, we have now used FRC_align (FRCBam) to compare our new assembly to the older MesAur1.0 (lines 282 – 284 and Supplementary Figure 1).

Section titled "Transcript and protein alignments and annotation comparisons": How many INDELs were identified in the alignments of RNA-seq transcripts to the BCM_Maur_2.0 assembly? Was this count different from those discovered in the short read assembly?

We have now added (lines 319 – 322) the number of RefSeq gene models that required sequence gap fills in BCM_Maur_2.0 (33 fills) as compared to MesAur1.0 (5,050 fills). This is clearly a substantial improvement.

Section titled "Interferon type 1 alpha gene cluster": Were there any gaps that spanned the gene cluster or flanked it?

We have added a statement to the final paragraph of the Results section (lines 359 - 362) indicating that the interferon type I alpha gene cluster in our new assembly lies within more than 12 Mbp of contiguous genomic sequence with the nearest flanking gaps located 2.66 Mbp proximal and 9.07 Mbp distal to the Ifne and Ifnb1 genes, respectively.

Reviewer #2

Harris et al. present a high-quality Syrian hamster assembly BCM_Maur_2.0 with ONT long reads, Illumina short reads and Bionano optical map. They performed comprehensive comparison between the published hamster assembly MesAur1.0 and the new assembly, and further performed preliminary analysis to the interferon alpha I cluster. The discussion summarized the study well and provided possible further research fields. The work can undoubtedly contribute to the evolution and long disease studies. The whole paper is clearly described and easy to read. Thus, I think the paper is worthy for publication.

We thank the reviewer for this positive appraisal.

Below are some minor questions and suggestions for the paper:
1. Why were gaps introduced after Pilon polishing, according to the number in Table 1?

We are afraid there is some confusion here and we apologize if the initial text and table was not clear. There were no gaps introduced by the Pilon polishing step, which only corrects errors in basepair calls. The Bionano optical mapping step did identify a number of mis-joins in the initial scaffolding and therefore we broke some candidate scaffolds, creating separation among them. Please see Table 1 and lines 202 – 204).

2. When performing the assembly comparisons, the authors showed BCM_Maur_2.0 has much less gap than MesAur1.0. Could the authors show the number of genes that is rescued from the gap? This could further prove the advantage of the new assembly.

We agree with the reviewer that the number of gene models rescued or completed through the new assembly is an important issue. We feel that the BUSCO analysis is our best approach to assessing the degree of improvement. We found that the proportion of missing genes drops from 5.1% in MesAur1.0 to 3.2% in BCM_Maur_2.0 while the proportion of complete BUSCO genes increases from 89.0% in the earlier assembly to 93.9% in the new one. This information and further related statistics are provided in Table 2.

3. The author wrote "The NCBI annotations characterize twelve of these genes as interferon alpha- 12-like with predicted coding sequences of 190 amino acids (Supplemental Table 1).", but in the table gene LOC101824534 encodes protein with only 183aa. I suggest rewording the sentence, for example, remove the length description. Besides, in the paper the author preliminarily studied the interferon alpha genes. It would be better to be careful about the pseudogenes in further analysis, to avoid false positive caused by ONT sequencing errors which are not corrected by the Illumina short reads with Pilon.

We thank the reviewer for these suggestions and have made these changes in the text (lines 352 – 353).

4. Gene name in Fig. 3 should be italic. And it's better to also show the ifn1 alpha cluster of MesAur1.0 in the plot as well for a clear comparison between the assemblies.

We have italicized gene names and have added the MesAur1.0 chromosome region to Figure 3.

5. Version of some softwares such as Pilon and Assemblytics are missing

We now indicate in the Genome Assembly section that we used Pilon version 1.23 (line 165) and in the Quality Assessment section that we used Assemblytics version 1.2.1 (line 212). We have checked other software tools and have included version numbers if they were missing in the first submission.

Close
