# Supplementary material for: Construction of a new chromosome-scale, long-read reference genome assembly for the Syrian hamster, Mesocricetus auratus
Source: Gigascience. 2022 May 28;11:giac039. doi: 10.1093/gigascience/giac039 (PMC9155146; doi:10.1093/gigascience/giac039)
Supplement: giac039_Response_to_Reviewer_Comments_Revision_1 [file giac039_response_to_reviewer_comments_revision_1.pdf]

Author's Response To Reviewer Comments

Close

Response to Reviews for GIGA-D-21-00197R1
“Construction of a new chromosome-scale, long read reference genome assembly for the Syrian hamster, Mesocricetus auratus”

We thank the editor and reviewers for their additional feedback and critiques of our manuscript. We have now performed additional analyses as suggested and made changes to the manuscript to address all the reviewers’ comments. The reviewers’ comments are in regular text, and our responses are in italics.

Reviewer #1

Summary: In this revision, the authors have addressed most of my major concerns with the manuscript. More details must be provided in two sections of the manuscript based on new details provided by the authors. However, these concerns could feasibly be addressed in revision.

Line 124: While the authors have provided an explanation for the sequencing of different target fragment length library preparations, I do not see any results that suggest that one particular preparation was more efficient than the others. This is particularly important given the prevalence of four experimental runs of varying dataset sizes that were uploaded to the cited Biosample accession on SRA. Currently, the metadata provided for that Biosample and its associated experiments is lacking, and one cannot easily distinguish which experiment resulted from different target length preparations. A discursive analysis is not required here, but a statement that provides limited data supporting the authors' preference for library prep is necessary.

We thank the reviewer for this suggestion. We have added a statement to the Results section of the manuscript that describes our current thinking regarding the optimal target length for DNA fragments to be used as input to Nanopore libraries (lines 234-237). Based on our experience in this work on hamster, as well as other projects, we find that a target fragment size of 15-20 kb performs best, and we now report this in the main text of our paper.

Line 301: I believe that the authors misinterpreted the comment on this section in my last review. I requested the proportion of sequence identity differences between assemblies due to INDELs, not assembly gaps. Residual INDELs are still a major problem in polished assemblies that may impact gene annotation.

We apologize for misunderstanding the original comment. Given that the two assemblies (MesAur1.0 and BCM_Maur_2.0) were produced using DNA from different hamsters, and that the number and allele frequencies of indel polymorphisms in this species are unknown, we calculated the percent of indels found in alignments of RefSeq transcripts to both assemblies. We feel this provides clear and meaningful information regarding the issue of residual indel errors in the assemblies, especially the new long read assembly. We have added the following statement to “Transcript and Protein Alignments and Annotation Comparisons” section of the paper (lines 308-310):

“Alignments of RefSeq transcripts showed a similar average percent indels in the BCM_Maur_2.0 (0.10%) and MesAur1.0 (0.11%) assemblies.”

Figure 1 caption: Given the new k-mer genome size estimation analysis provided by the authors, it does not make sense to use the total length of the MesAur1.0 assembly here. I believe that the authors should choose a genome size estimate that seems most reasonable (from the two options provided) and then use that as the basis for NG50 comparisons. Otherwise, are they conceding that the MesAur1.0 assembly size is the full length of the Syrian Hamster sequence-accessible genome?

We have now regenerated Figure 1 using the SGA preqc genome length estimate of 2.57 Gbp. We have also changed the figure caption to state that we used the SGA preqc genome length estimate (lines 461-462), and added this to the text of the Results section (lines 277-278).

Reviewer #2

The authors have resolved most of my comments. However, I am still confused about the gap in the Pilon step from the information in Table 1. In the table, I could read that the assembly length of "Flye + Pilon" is 2,383,228,608 bp, and the ungapped legnth is 2,383,226,373 bp, so the gap length is 2,383,228,608 - 2,383,226,373 = 2,235 bp. Because in the "Flye" version the assembly length is equal to the ungapped legnth, this means that gaps are introduced after Pilon correction.

Here are numbers in question from Table 1 along with the differences between the assembly length and the ungapped length:

Parameter Flye Flye + Pilon
Assembly length (bp) 2,381,258,546 2,383,228,608
Ungapped length (bp) 2,381,254,546 2,383,226,373
Difference (bp) 4,000 2,235

The Flye assembly length and ungapped length are in fact different by 4000bp, not equal. This is because Flye inserts standardized 100bp gaps during the assembly process. The Flye + Pilon assembly length and ungapped length are different by 2235bp as stated by Reviewer #2. Thus, the Flye + Pilon assembly has smaller gap length than the initial Flye assembly because the Pilon polishing either closed or reduced the size of some gaps.

We thank the reviewers for their feedback and hope that these revisions will now make our manuscript acceptable for publication.

Close
