# Supplementary material for: Construction of a new chromosome-scale, long-read reference genome assembly for the Syrian hamster, Mesocricetus auratus
Source: Gigascience. 2022 May 28;11:giac039. doi: 10.1093/gigascience/giac039 (PMC9155146; doi:10.1093/gigascience/giac039)
Supplement: giac039_Reviewer_1_Report_Original_Submission — Derek Bickhart -- 8/16/2021 Reviewed [file giac039_reviewer_1_report_original_submission.pdf]

Reviewer Report

Title: Construction of a new chromosome-scale, long-read reference genome assembly for the Syrian hamster, Mesocricetus auratus

Version: Original Submission **Date:** 8/16/2021

Reviewer name: Derek Bickhart

Reviewer Comments to Author:

Summary: In this manuscript, Harris et al. detail the methods they used to create a new reference genome for the Syrian hamster, which is an important model for respiratory disease pathogens. They used several different sequencing technologies to generate the contigs and scaffolds for their new assembly, and achieved a relatively continuous end product. The analysis is suitable for the "genome report" style format (with one omission detailed below in my comments); however, the manuscript suffers from some awkward phrasing and grammar errors in the results and methods. I list my comments below in the relative order in which I encountered them in the manuscript. Since the authors did not provide line numbers in their submission, I provide my comments as a block listing of questions/suggestions/critiques.

Section titled "oxford nanopore long-read sequencing": The description of the shearing is awkward. I recommend revising the first sentence to state that the genomic DNA isolates were sheared to three lengths (without providing these lengths in the sentence). In subsequent sentences, provide the lengths in situ with the methods used to prepare them. Also, it is unclear why three different fragment lengths were used here for oxford nanopore sequencing. Given that these fragment lengths are relatively similar in size (e.g. not disparate lengths similar to recent ultra-long nanopore read preps of >100kb), it would be very helpful to the reader if justification was given for this approach.

Section titled "Genome assembly": This entire paragraph is awkwardly phrased with numerous past- or present-tense changes. Additionally, the reference to the Pilon polisher needs to be cited, and details need to be provided on what settings were used for Pilon polishing (it is often recommended to correct only indels and to omit gap-filling) and how many iterations of polishing were used. Details are missing on how BioNano optical maps were generated, and what DNA was used as input in the process. Also, what software was used to compare BioNano optical maps, and with what settings? Finally, it appears that the RNA-seq data used by NCBI for annotation was used in another study. Citation to that study would be required so that the reader is aware that the data resulted from different individuals other than the reference individual sequenced in this analysis.

Section titled "Assembly Comparisons": What is the expected c-value of the Syrian Hamster genome? Also, what is the karyotype count? Are any of the chromosomes metacentric or acrocentric? Were any satellite regions identified and annotated in this assembly? Finally, I would have preferred that assembly comparisons be conducted with feature response curves, such as those produced by the program "FRC_align" as this provides a useful metric to assess assembly "correctness" by length.

Section titled "Transcript and protein alignments and annotation comparisons": How many INDELs were identified in the alignments of RNA-seq transcripts to the BCM_Maur_2.0 assembly? Was this count

different from those discovered in the short read assembly?
Section titled "Interferon type 1 alpha gene cluster": Were there any gaps that spanned the gene cluster or flanked it?

Level of Interest

Please indicate how interesting you found the manuscript: Choose an item.

Quality of Written English

Please indicate the quality of language in the manuscript: Choose an item.

Declaration of Competing Interests

Please complete a declaration of competing interests, considering the following questions:

- Have you in the past five years received reimbursements, fees, funding, or salary from an organisation that may in any way gain or lose financially from the publication of this manuscript, either now or in the future?
- Do you hold any stocks or shares in an organisation that may in any way gain or lose financially from the publication of this manuscript, either now or in the future?
- Do you hold or are you currently applying for any patents relating to the content of the manuscript?
- Have you received reimbursements, fees, funding, or salary from an organization that holds or has applied for patents relating to the content of the manuscript?
- Do you have any other financial competing interests?
- Do you have any non-financial competing interests in relation to this paper?

If you can answer no to all of the above, write 'I declare that I have no competing interests' below. If your reply is yes to any, please give details below.

I declare that I have no competing interests

I agree to the open peer review policy of the journal. I understand that my name will be included on my report to the authors and, if the manuscript is accepted for publication, my named report including any attachments I upload will be posted on the website along with the authors' responses. I agree for my report to be made available under an Open Access Creative Commons CC-BY license (http://creativecommons.org/licenses/by/4.0/). I understand that any comments which I do not wish to be included in my named report can be included as confidential comments to the editors, which will not be published.

Choose an item.

To further support our reviewers, we have joined with Publons, where you can gain additional credit to further highlight your hard work (see: https://publons.com/journal/530/gigascience). On publication of this paper, your review will be automatically added to Publons, you can then choose whether or not to claim your Publons credit. I understand this statement.

Yes Choose an item.
