# Supplementary material for: Construction of a new chromosome-scale, long-read reference genome assembly for the Syrian hamster, Mesocricetus auratus
Source: Gigascience. 2022 May 28;11:giac039. doi: 10.1093/gigascience/giac039 (PMC9155146; doi:10.1093/gigascience/giac039)
Supplement: giac039_Reviewer_1_Report_Revision_1 — Derek Bickhart -- 11/12/2021 Reviewed [file giac039_reviewer_1_report_revision_1.pdf]

Reviewer Report

Title: Construction of a new chromosome-scale, long-read reference genome assembly for the Syrian hamster, Mesocricetus auratus

Version: Revision 1 **Date:** 11/12/2021

Reviewer name: Derek Bickhart

Reviewer Comments to Author:

Summary: In this revision, the authors have addressed most of my major concerns with the manuscript. More details must be provided in two sections of the manuscript based on new details provided by the authors. However, these concerns could feasibly be addressed in revision.
Line 124: While the authors have provided an explanation for the sequencing of different target fragment length library preparations, I do not see any results that suggest that one particular preparation was more efficient than the others. This is particularly important given the prevalence of four experimental runs of varying dataset sizes that were uploaded to the cited Biosample accession on SRA. Currently, the metadata provided for that Biosample and its associated experiments is lacking, and one cannot easily distinguish which experiment resulted from different target length preparations. A discursive analysis is not required here, but a statement that provides limited data supporting the authors' preference for library prep is necessary.
Line 301: I believe that the authors misinterpreted the comment on this section in my last review. I requested the proportion of sequence identity differences between assemblies due to INDELs, not assembly gaps. Residual INDELs are still a major problem in polished assemblies that may impact gene annotation.
Figure 1 caption: Given the new k-mer genome size estimation analysis provided by the authors, it does not make sense to use the total length of the MesAur1.0 assembly here. I believe that the authors should choose a genome size estimate that seems most reasonable (from the two options provided) and then use that as the basis for NG50 comparisons. Otherwise, are they conceding that the MesAur1.0 assembly size is the full length of the Syrian Hamster sequence-accessible genome?

Level of Interest

Please indicate how interesting you found the manuscript: Choose an item.

Quality of Written English

Please indicate the quality of language in the manuscript: Choose an item.

Declaration of Competing Interests

Please complete a declaration of competing interests, considering the following questions:

- Have you in the past five years received reimbursements, fees, funding, or salary from an organisation that may in any way gain or lose financially from the publication of this manuscript, either now or in the future?
- Do you hold any stocks or shares in an organisation that may in any way gain or lose financially from the publication of this manuscript, either now or in the future?
- Do you hold or are you currently applying for any patents relating to the content of the manuscript?
- Have you received reimbursements, fees, funding, or salary from an organization that holds or has applied for patents relating to the content of the manuscript?
- Do you have any other financial competing interests?
- Do you have any non-financial competing interests in relation to this paper?

If you can answer no to all of the above, write 'I declare that I have no competing interests' below. If your reply is yes to any, please give details below.

I declare that I have no competing interests.

I agree to the open peer review policy of the journal. I understand that my name will be included on my report to the authors and, if the manuscript is accepted for publication, my named report including any attachments I upload will be posted on the website along with the authors' responses. I agree for my report to be made available under an Open Access Creative Commons CC-BY license (http://creativecommons.org/licenses/by/4.0/). I understand that any comments which I do not wish to be included in my named report can be included as confidential comments to the editors, which will not be published.

Choose an item.

To further support our reviewers, we have joined with Publons, where you can gain additional credit to further highlight your hard work (see: https://publons.com/journal/530/gigascience). On publication of this paper, your review will be automatically added to Publons, you can then choose whether or not to claim your Publons credit. I understand this statement.

Yes Choose an item.
