# Supplementary material for: Construction of a new chromosome-scale, long-read reference genome assembly for the Syrian hamster, Mesocricetus auratus
Source: Gigascience. 2022 May 28;11:giac039. doi: 10.1093/gigascience/giac039 (PMC9155146; doi:10.1093/gigascience/giac039)
Supplement: giac039_Reviewer_2_Report_Original_Submission — Yang Zhou -- 8/25/2021 Reviewed [file giac039_reviewer_2_report_original_submission.pdf]

Reviewer Report

Title: Construction of a new chromosome-scale, long-read reference genome assembly for the Syrian hamster, Mesocricetus auratus

Version: Original Submission **Date:** 8/25/2021

Reviewer name: Yang Zhou

Reviewer Comments to Author:

Harris et al. present a high-quality Syrian hamster assembly BCM_Maur_2.0 with ONT long reads, Illumina short reads and Bionano optical map. They performed comprehensive comparison between the published hamster assembly MesAur1.0 and the new assembly, and further performed preliminary analysis to the interferon alpha I cluster. The discussion summarized the study well and provided possible further research fields. The work can undoubtedly contribute to the evolution and long disease studies. The whole paper is clearly described and easy to read. Thus, I think the paper is worthy for publication.

Below are some minor questions and suggestions for the paper:

1. Why were gaps introduced after Pilon polishing, according to the number in Table 1?
2. When performing the assembly comparisons, the authors showed BCM_Maur_2.0 has much less gap than MesAur1.0. Could the authors show the number of genes that is rescued from the gap? This could further prove the advantage of the new assembly.
3. The author wrote "The NCBI annotations characterize twelve of these genes as interferon alpha- 12-like with predicted coding sequences of 190 amino acids (Supplemental Table 1).", but in the table gene LOC101824534 encodes protein with only 183aa. I suggest rewording the sentence, for example, remove the length description. Besides, in the paper the author preliminarily studied the interferon alpha genes. It would be better to be careful about the pseudogenes in further analysis, to avoid false positive caused by ONT sequencing errors which are not corrected by the Illumina short reads with Pilon.
4. Gene name in Fig. 3 should be italic. And it's better to also show the ifn1 alpha cluster of MesAur1.0 in the plot as well for a clear comparison between the assemblies.
5. Version of some softwares such as Pilon and Assemblytics are missing

Level of Interest

Please indicate how interesting you found the manuscript: Choose an item.

Quality of Written English

Please indicate the quality of language in the manuscript: Choose an item.

Declaration of Competing Interests

Please complete a declaration of competing interests, considering the following questions:

- Have you in the past five years received reimbursements, fees, funding, or salary from an organisation that may in any way gain or lose financially from the publication of this manuscript, either now or in the future?
- Do you hold any stocks or shares in an organisation that may in any way gain or lose financially from the publication of this manuscript, either now or in the future?
- Do you hold or are you currently applying for any patents relating to the content of the manuscript?
- Have you received reimbursements, fees, funding, or salary from an organization that holds or has applied for patents relating to the content of the manuscript?
- Do you have any other financial competing interests?
- Do you have any non-financial competing interests in relation to this paper?

If you can answer no to all of the above, write 'I declare that I have no competing interests' below. If your reply is yes to any, please give details below.

I declare that I have no competing interests

I agree to the open peer review policy of the journal. I understand that my name will be included on my report to the authors and, if the manuscript is accepted for publication, my named report including any attachments I upload will be posted on the website along with the authors' responses. I agree for my report to be made available under an Open Access Creative Commons CC-BY license (http://creativecommons.org/licenses/by/4.0/). I understand that any comments which I do not wish to be included in my named report can be included as confidential comments to the editors, which will not be published.

Choose an item.

To further support our reviewers, we have joined with Publons, where you can gain additional credit to further highlight your hard work (see: https://publons.com/journal/530/gigascience). On publication of this paper, your review will be automatically added to Publons, you can then choose whether or not to claim your Publons credit. I understand this statement.

Yes Choose an item.
